# Supplementary material for: MYSM1 acts as a novel co-activator of ERα to confer antiestrogen resistance in breast cancer
Source: EMBO Mol Med. 2023 Dec 15;16(1):4. doi: 10.1038/s44321-023-00003-z (PMC10883278; doi:10.1038/s44321-023-00003-z)
Supplement: Supplementary file 1 — Appendix [file 44321_2023_3_MOESM1_ESM.pdf]

Appendix Table of Contents

1. Appendix Fig S1
2. Appendix Fig S1 legends
3. Appendix Fig S2
4. Appendix Fig S2 legends

Appendix Fig S1

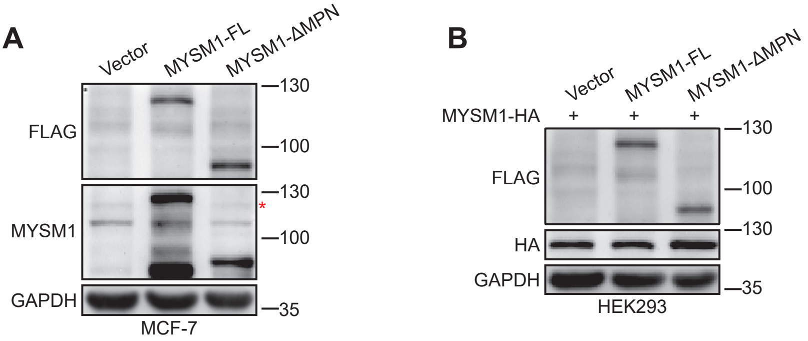

Appendix Figure S1. MYSM1 has no effect on its own protein expression

A. Western blot detecting endogenous MYSM1 expression in MCF-7 cells transfected with PcDNA3.1/MYSM1/MYSM1-ΔMPN expression plasmids.

B. Immunoblot of exogenous MYSM1 expression using the HA antibodies in PcDNA3.1/MYSM1/MYSM1-ΔMPN overexpressed HEK293 cells.

Appendix Fig S2

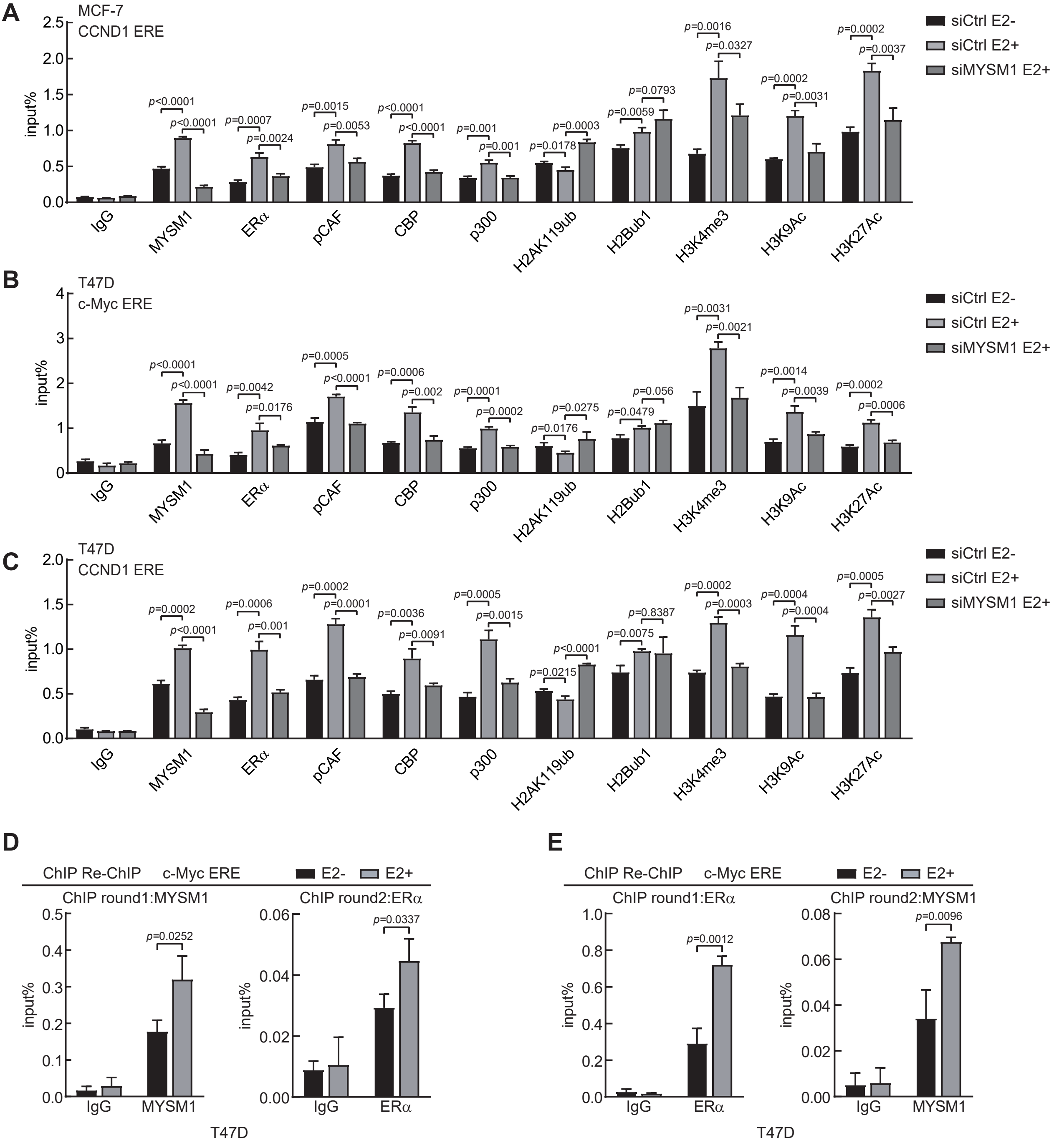

Appendix Figure S2. MYSM1 facilitates the recruitment of ERα and HAT complex at the *cis* elements of ERα target genes in MCF-7 cells

A. ChIP assays via designated antibodies to elaborate the influence of MYSM1 regarding to ERα recruitment and relative histone modifications on *CCND1* ERE in MCF-7 cells (mean ± SD; Student *t*-test).

B, C. ChIP analysis was conducted with specific antibodies in MYSM1-deficiency T47D cells on the promoter region of *c-Myc* (B) or *CCND1* (C) with or without E2 (mean ± SD; Student *t*-test).

D, E. ChIP re-ChIP assay was performed with the indicated antibodies in T47D cells (mean ± SD; Student *t*-test).
