# Supplementary material for: MYSM1 acts as a novel co-activator of ERα to confer antiestrogen resistance in breast cancer
Source: EMBO Mol Med. 2023 Dec 15;16(1):4. doi: 10.1038/s44321-023-00003-z (PMC10883278; doi:10.1038/s44321-023-00003-z)
Supplement: Supplementary file 3 — Table EV1 [file 44321_2023_3_MOESM3_ESM.docx]

**Table EV1. Primers used for MYSM1 plasmids construction in this study**

| Name | Primer sequence |
| --- | --- |
| MYSM1-FL-F’ | GACGATGACAAGCTTATGGCGGCTGAAGAGGC |
| MYSM1-FL-R’ | TGCCACCCGGGATCCTCACATTAACAATTCCTTTGTACA |
| MYSM1-ΔMPN-R’ | TGCCACCCGGGATCCTCACTGAAATGGCTCCTGCTTTT |
| MYSM1-ΔSANT-F’ | GACGATGACAAGCTTAAATGCGGTCTGGATAAAGAAA |
| MYSM1-ΔSWIRM-1R’ | TTGTGGCTCTTCTTCCTCATGGCTTTCCTC |
| MYSM1-ΔSWIRM-2F’ | GAAGAAGAGCCACAAACAGTTGACAAAGTACG |
