# Supplementary material for: MYSM1 acts as a novel co-activator of ERα to confer antiestrogen resistance in breast cancer
Source: EMBO Mol Med. 2023 Dec 15;16(1):4. doi: 10.1038/s44321-023-00003-z (PMC10883278; doi:10.1038/s44321-023-00003-z)
Supplement: Supplementary file 4 — Table EV2 [file 44321_2023_3_MOESM4_ESM.docx]

**Table EV2. Primers used for qPCR**

| Name | Sense(F’) | Anti-sense(R’) |
| --- | --- | --- |
| MYSM1 m | ATTAGCCCAGATGGCTCTTATC | GCTCAGAGTCTTCCTCATACAC |
| ESR1 m | GACTGCACTTGCTCCCGT | CCACTTCGTAGCATTTGCGG |
| c-Myc m | CGTCCTCGGATTCTCTGCTC | GCTGGTGCATTTTCGGTTGT |
| FOXC1 m | AGTCAGCTTGCTTTGAGGCTA | AGGCATCACCGTGGTAAGAC |
| VEGF m | GTCTTGACTCTACTCCACCCC | CTCGGTACTGACATCGCTCC |
| CCND1 m | CTGATTGGACAGGCATGGGT | GTGCCTGGAAGTCAACGGTA |
| E2F1 m | CAGAGCAGATGGTTATGGTGAT | AGATGATGGTGGTGGTGACA |
| GREB1 m | ATGGAGGACCTGGAGCAGAT | ACAGTGCTACTCACAAGATCCC |
| TFF1 m | TCCCCTGGTGCTTCTATCCT | GGACTAATCACCGTGCTGGG |
| c-Myc e | CATGAAAGGTGAAGCGGAAATAC | TGAAGGAGCAGGTGAAACG |
| CCND1 e | CGTCTTTTCCCACTGACACA | CCCCTCCCCAGAAGAAAATA |
| E2F1 e | ACGACTCAGTCTCAGTTTTAGC | GTATACAGGCCCAGAGTCAC |
| GREB1 e | GCTAACCATGCTGCAAATGA | ACACAGTCAGGGCAAAGGAC |
| TFF1 e | AGGGGATGTGTGTGAGAAGG | GCTTCGAGACAGTGGGAGTC |

*m represents mRNA expression and e represents eRNA expression
