# Supplementary material for: MYSM1 acts as a novel co-activator of ERα to confer antiestrogen resistance in breast cancer
Source: EMBO Mol Med. 2023 Dec 15;16(1):4. doi: 10.1038/s44321-023-00003-z (PMC10883278; doi:10.1038/s44321-023-00003-z)
Supplement: Supplementary file 5 — Table EV3 [file 44321_2023_3_MOESM5_ESM.docx]

**Table EV3. Primers of ChIP used for qPCR**

| Name | Sense(F’) | Anti-sense(R’) |
| --- | --- | --- |
| c-Myc p | TTTTTGTGCATGACCGCATTTC | ACCGGACTTCCTAAAAGGGG |
| VEGF p | CCTTTGCCTTGCTCTGTCAC | TGTTGTGCTGTTGTCCTCCA |
| CCND1 p | CGGAATTCGCTTTCTCCTGA | GCGGATTCTGGGCTCATTTA |
| TFF1 p | GGCCATCTCTCACTATGAATCACTTCTGC | GGCAGGCTCTGTTTGCTTAAAGAGCG |
| GREB1 p | GCTGACCTTGTGGTAGGCAC | CAGGGGCTGACAACTGAAAT |
| c-Myc e | GATGGGACTTGAACACCAGC | TTGTCTTTGGCCATGGTGGG |
| CCND1 e | TCACTGACACTGCATTTGCC | GCAAGTGGGGATGTGGTATGT |
| E2F1 e | ATAAGAACCCACTGTGGTGGC | TTCAACCTGAATGGCCCCTG |
| GREB1 e | AGTGGCCAGTGTGTAAGTGA | ATTTCCCAGTCCTTTTGCCA |
| TFF1 e | GTGGAAGCTGGGTCCTAGAAA | CCTATGGTTGGGGGTTTCCTG |

*p represents the promoter region and e represents the enhancer region
