# Supplementary figures and images for: MYSM1 acts as a novel co-activator of ERα to confer antiestrogen resistance in breast cancer
Source: EMBO Mol Med. 2023 Dec 15;16(1):4. doi: 10.1038/s44321-023-00003-z (PMC10883278; doi:10.1038/s44321-023-00003-z)

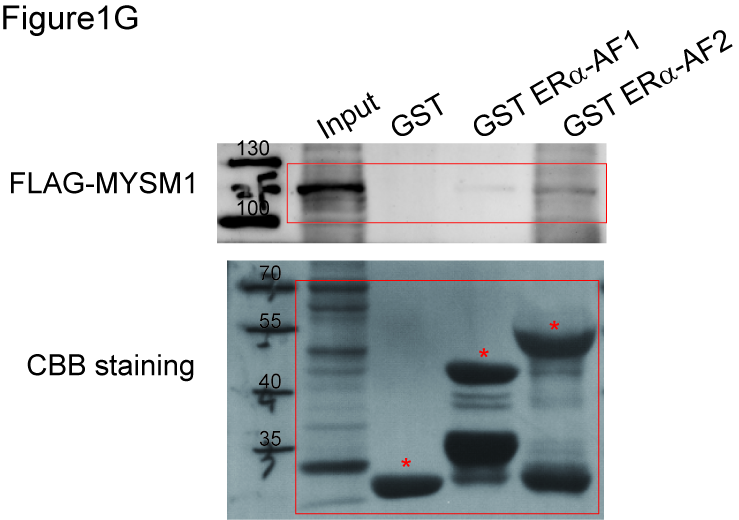

Supplement: Supplementary file 6 — Source Data Fig. 1 [file 44321_2023_3_MOESM6_ESM.zip › Figure 1/Fig 1G-Image data.tif]

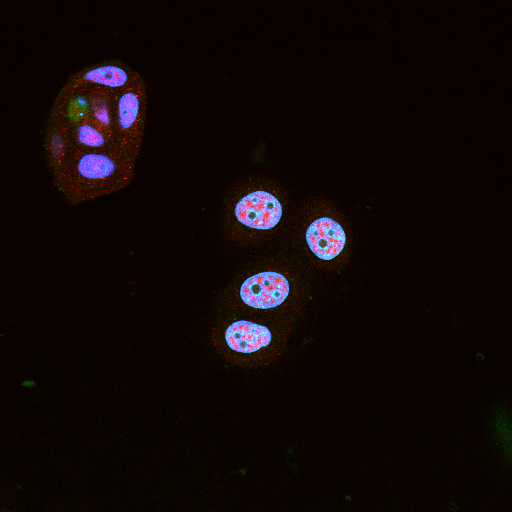

Supplement: Supplementary file 6 — Source Data Fig. 1 [file 44321_2023_3_MOESM6_ESM.zip › Figure 1/Fig 1I-Image data E2+.tif]

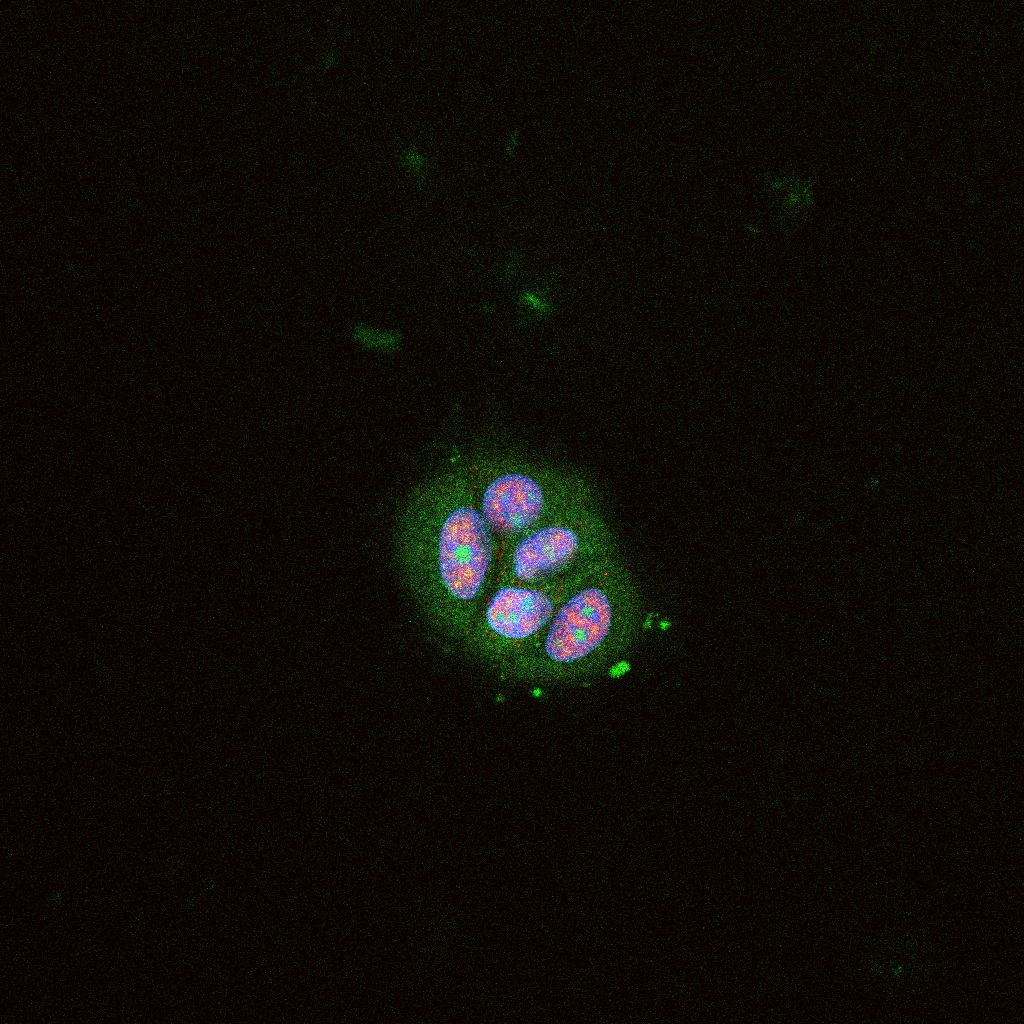

Supplement: Supplementary file 6 — Source Data Fig. 1 [file 44321_2023_3_MOESM6_ESM.zip › Figure 1/Fig 1I-Image data E2-.tif]

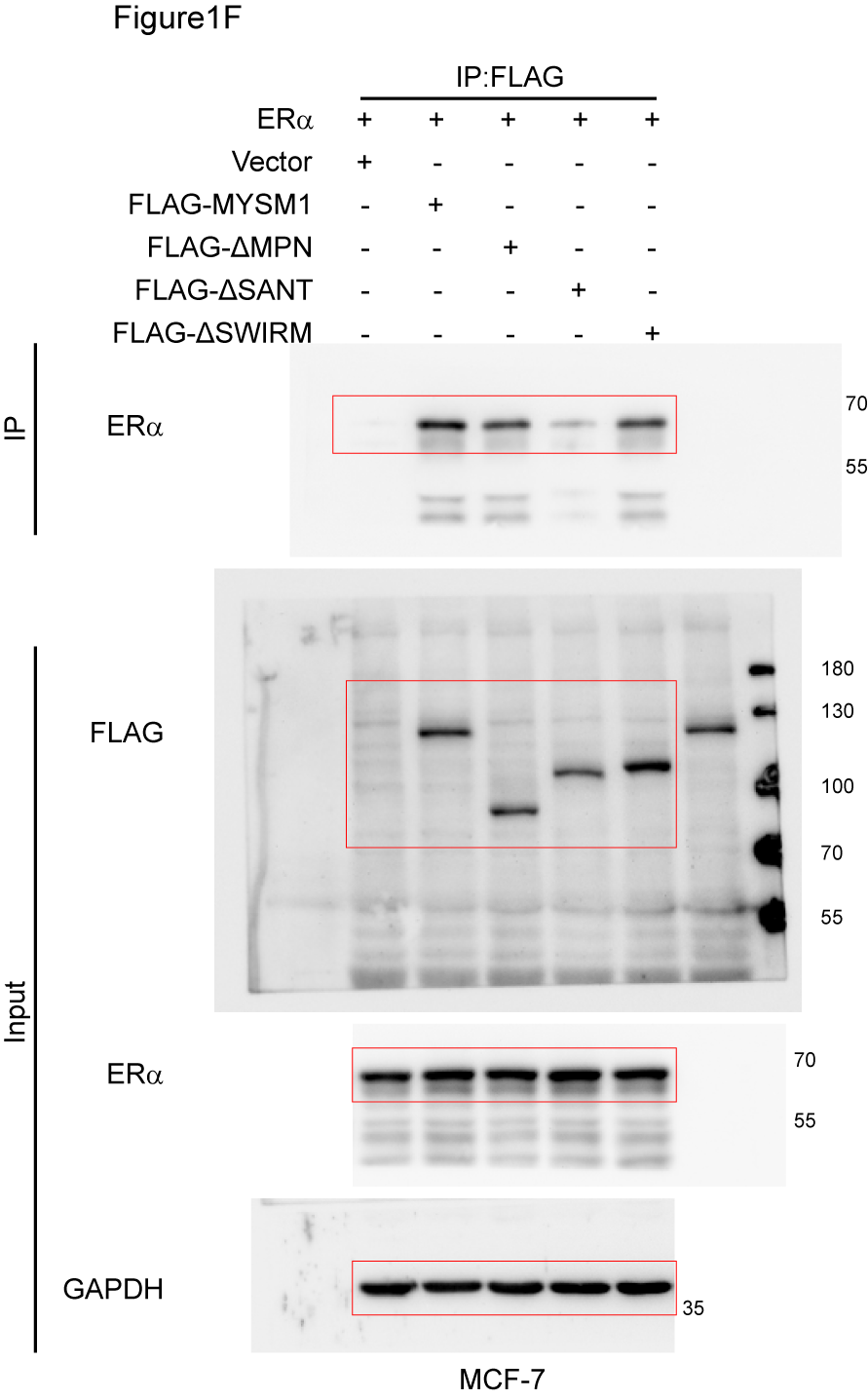

Supplement: Supplementary file 6 — Source Data Fig. 1 [file 44321_2023_3_MOESM6_ESM.zip › Figure 1/Fig 1F-Image data.tif]

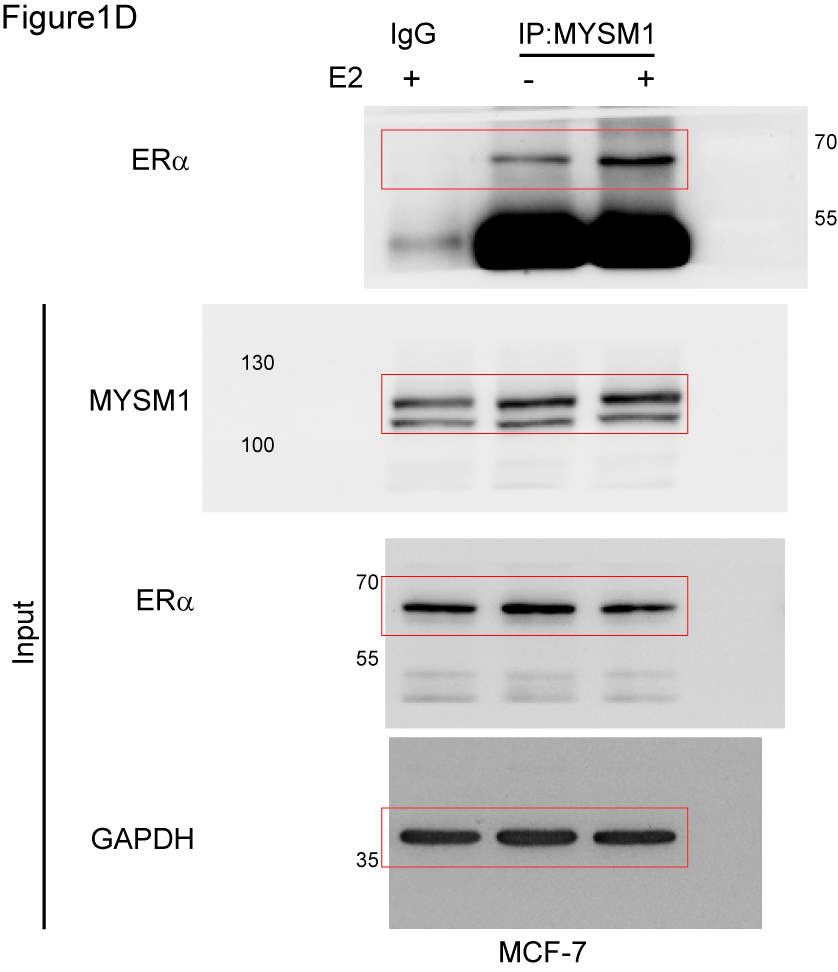

Supplement: Supplementary file 6 — Source Data Fig. 1 [file 44321_2023_3_MOESM6_ESM.zip › Figure 1/Fig 1D-Image data.tif]

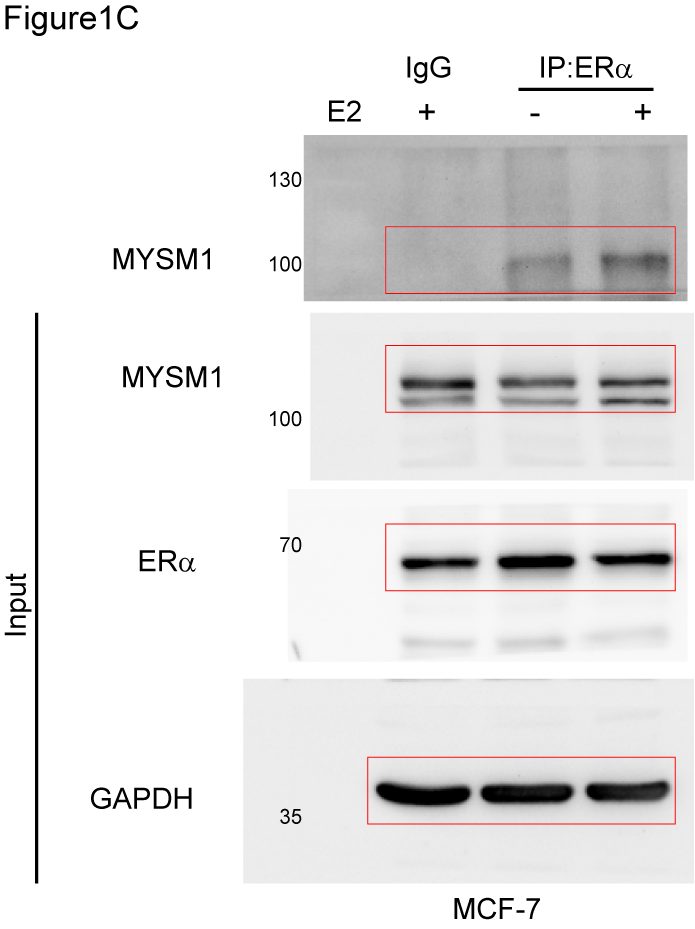

Supplement: Supplementary file 6 — Source Data Fig. 1 [file 44321_2023_3_MOESM6_ESM.zip › Figure 1/Fig 1C-Image data.tif]

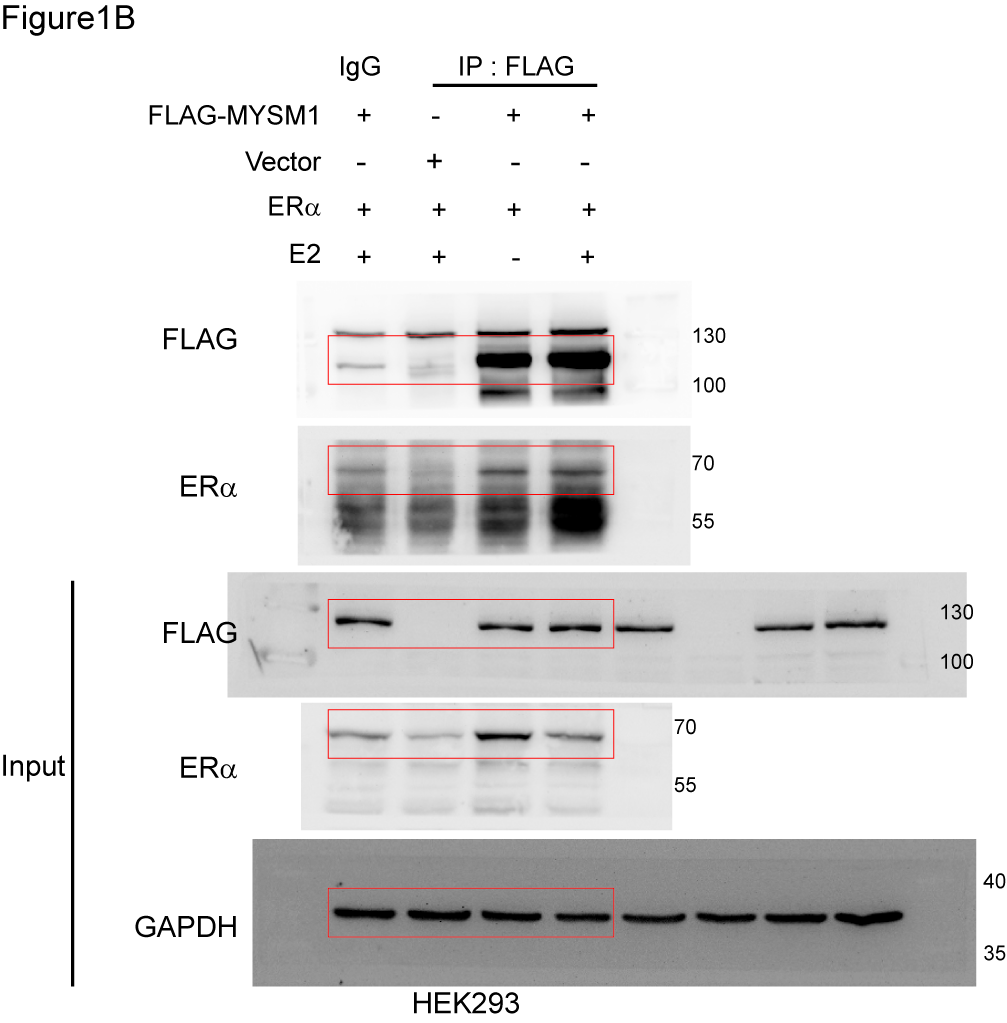

Supplement: Supplementary file 6 — Source Data Fig. 1 [file 44321_2023_3_MOESM6_ESM.zip › Figure 1/Fig 1B-Image data.tif]

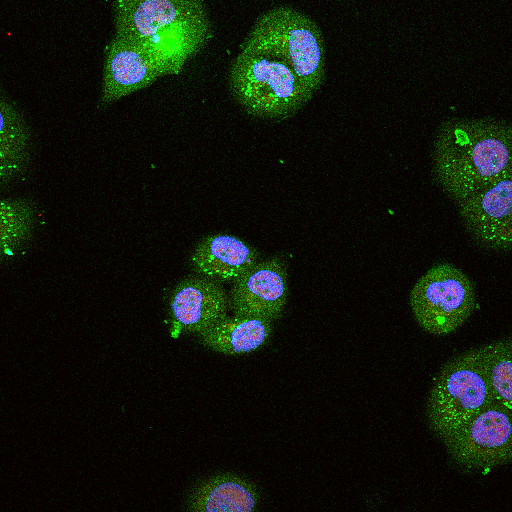

Supplement: Supplementary file 6 — Source Data Fig. 1 [file 44321_2023_3_MOESM6_ESM.zip › Figure 1/Fig 1H-Image data E2-.tif]

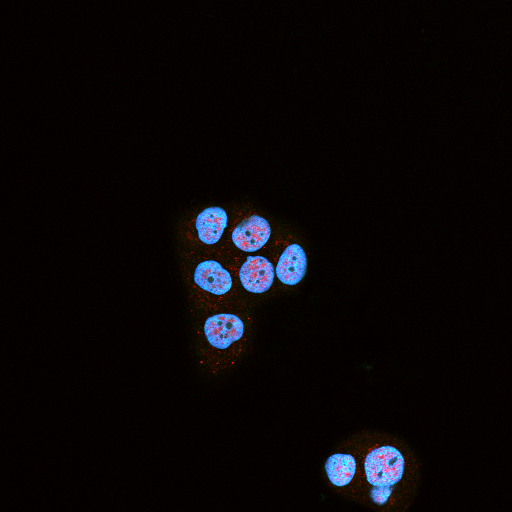

Supplement: Supplementary file 6 — Source Data Fig. 1 [file 44321_2023_3_MOESM6_ESM.zip › Figure 1/Fig 1H-Image data E2+.tif]

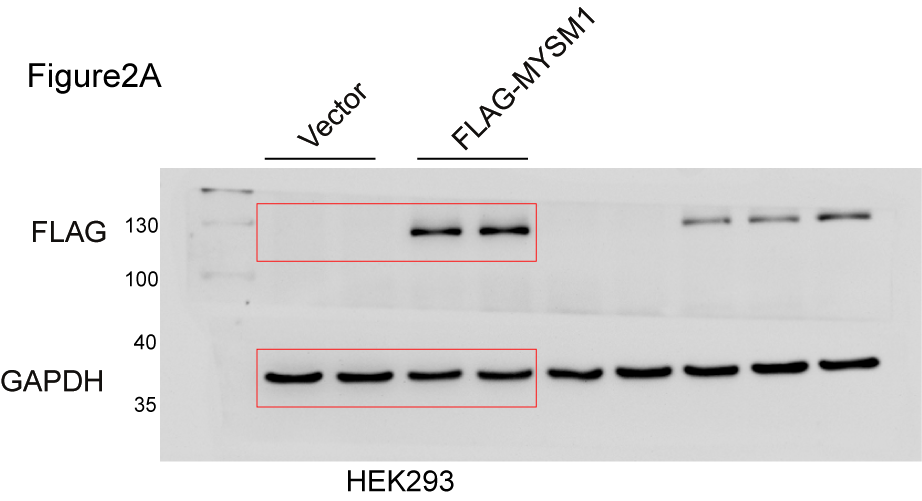

Supplement: Supplementary file 7 — Source Data Fig. 2 [file 44321_2023_3_MOESM7_ESM.zip › Figure 2/Fig 2A-Image data.tif]

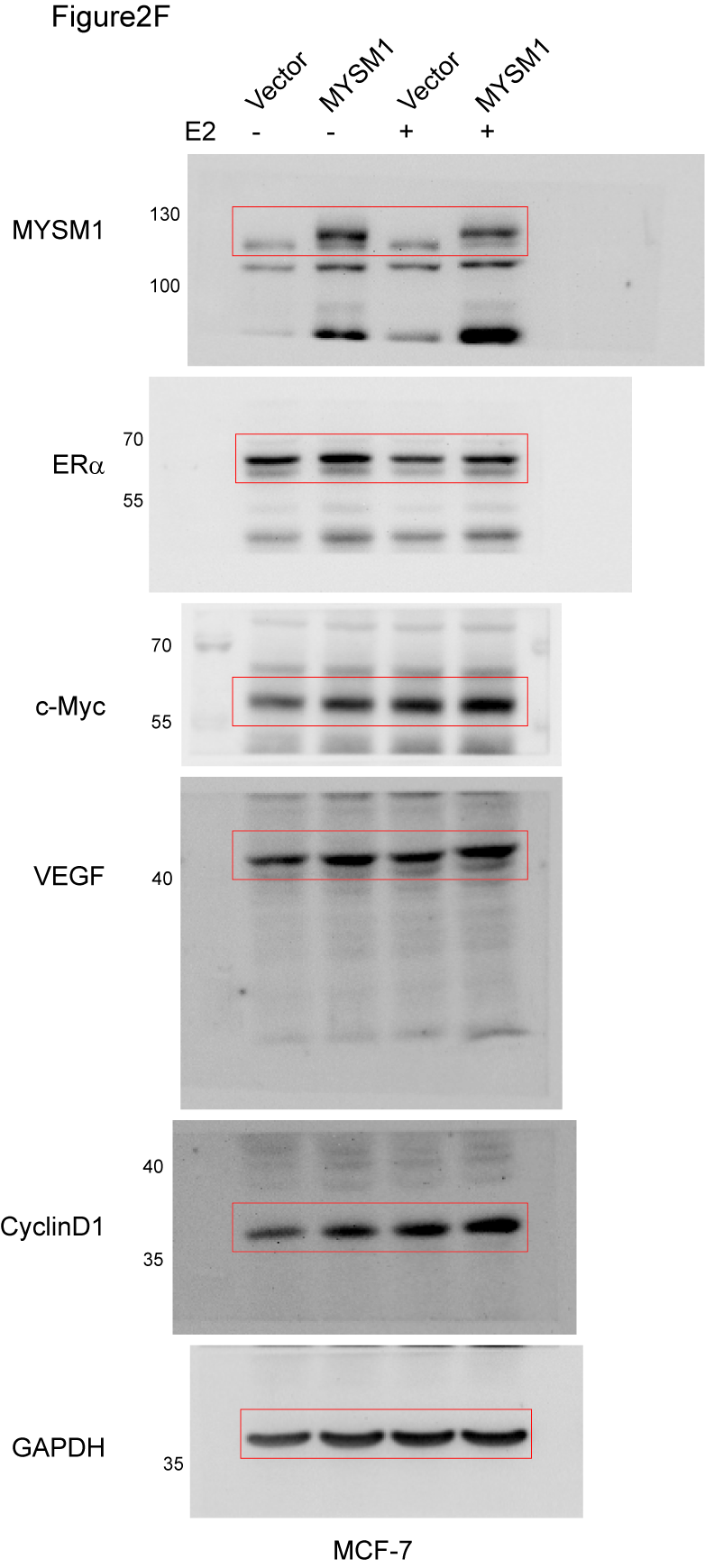

Supplement: Supplementary file 7 — Source Data Fig. 2 [file 44321_2023_3_MOESM7_ESM.zip › Figure 2/Fig 2F-Image data.tif]

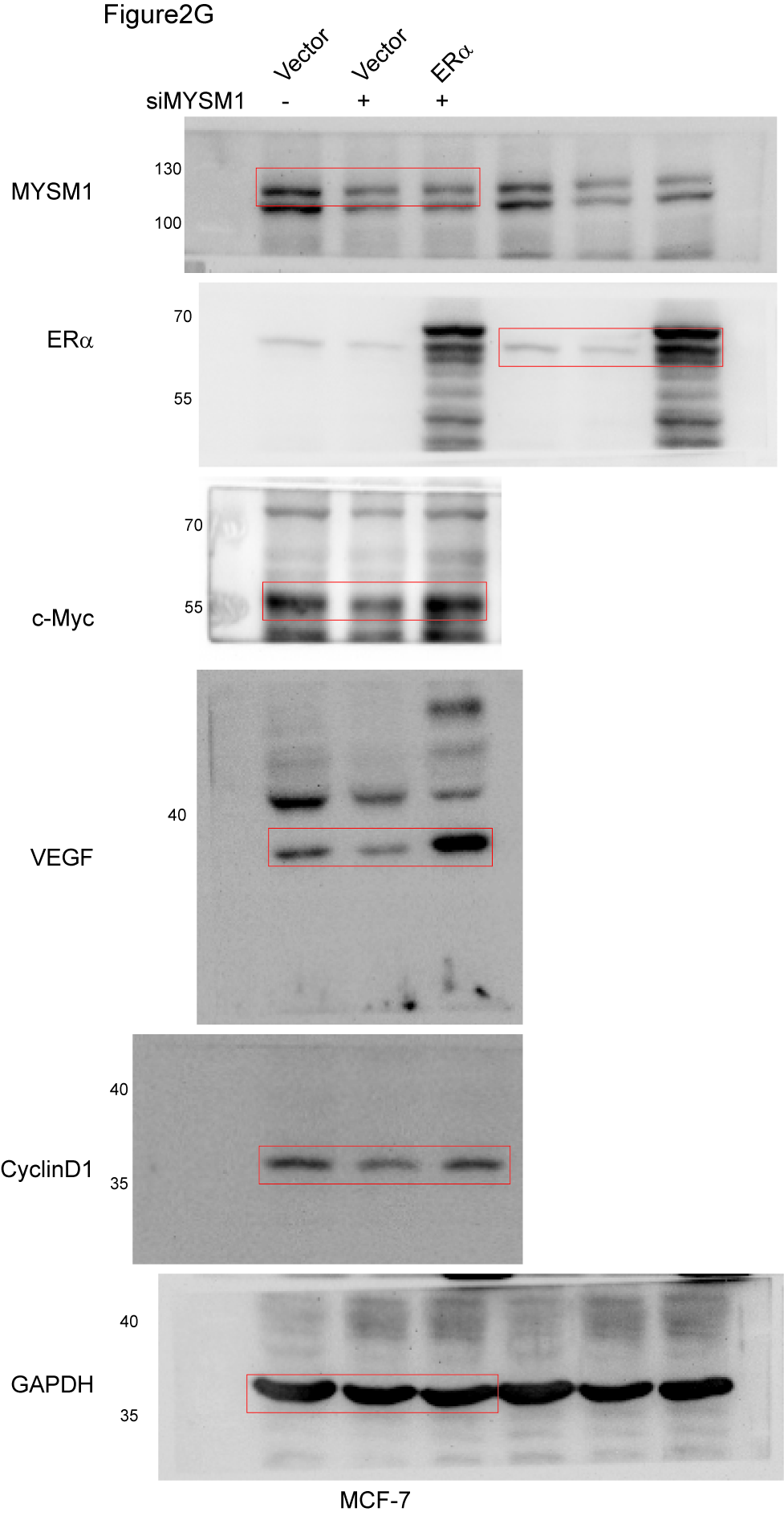

Supplement: Supplementary file 7 — Source Data Fig. 2 [file 44321_2023_3_MOESM7_ESM.zip › Figure 2/Fig 2G-Image data.tif]

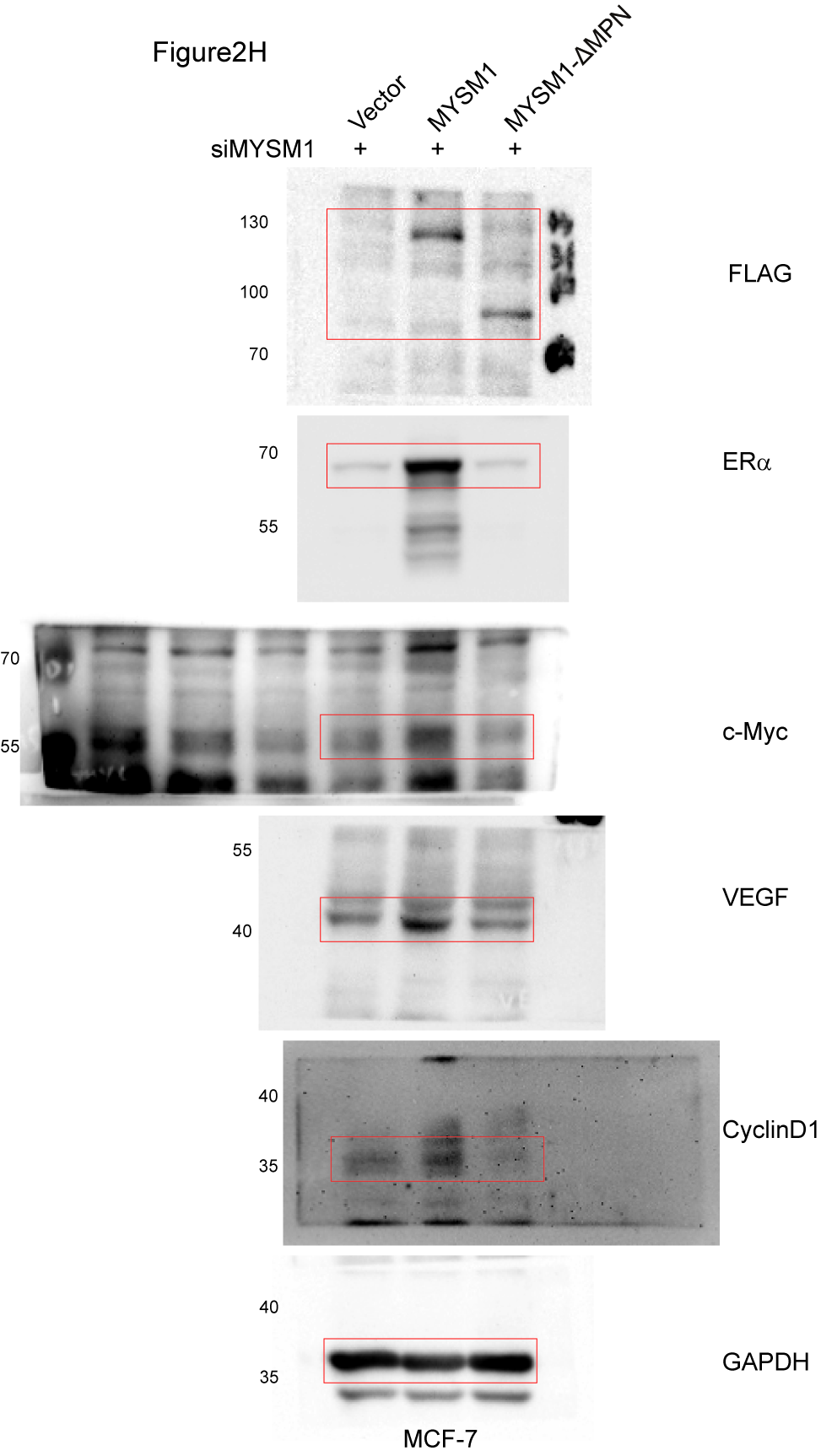

Supplement: Supplementary file 7 — Source Data Fig. 2 [file 44321_2023_3_MOESM7_ESM.zip › Figure 2/Fig 2H-Image data.tif]

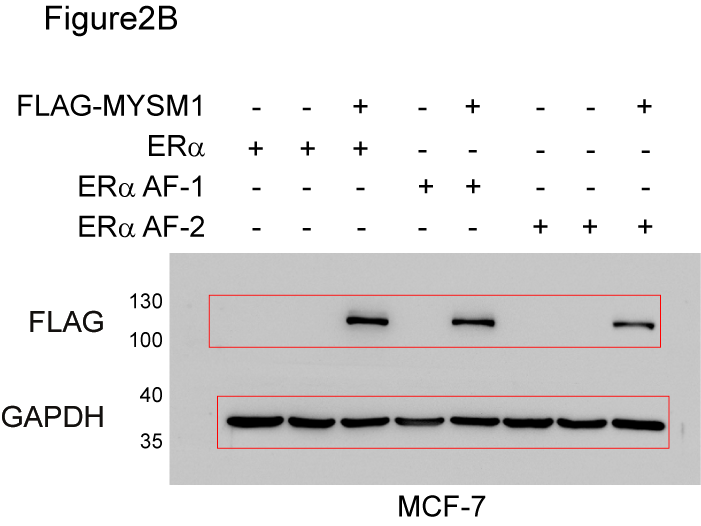

Supplement: Supplementary file 7 — Source Data Fig. 2 [file 44321_2023_3_MOESM7_ESM.zip › Figure 2/Fig 2B-Image data.tif]

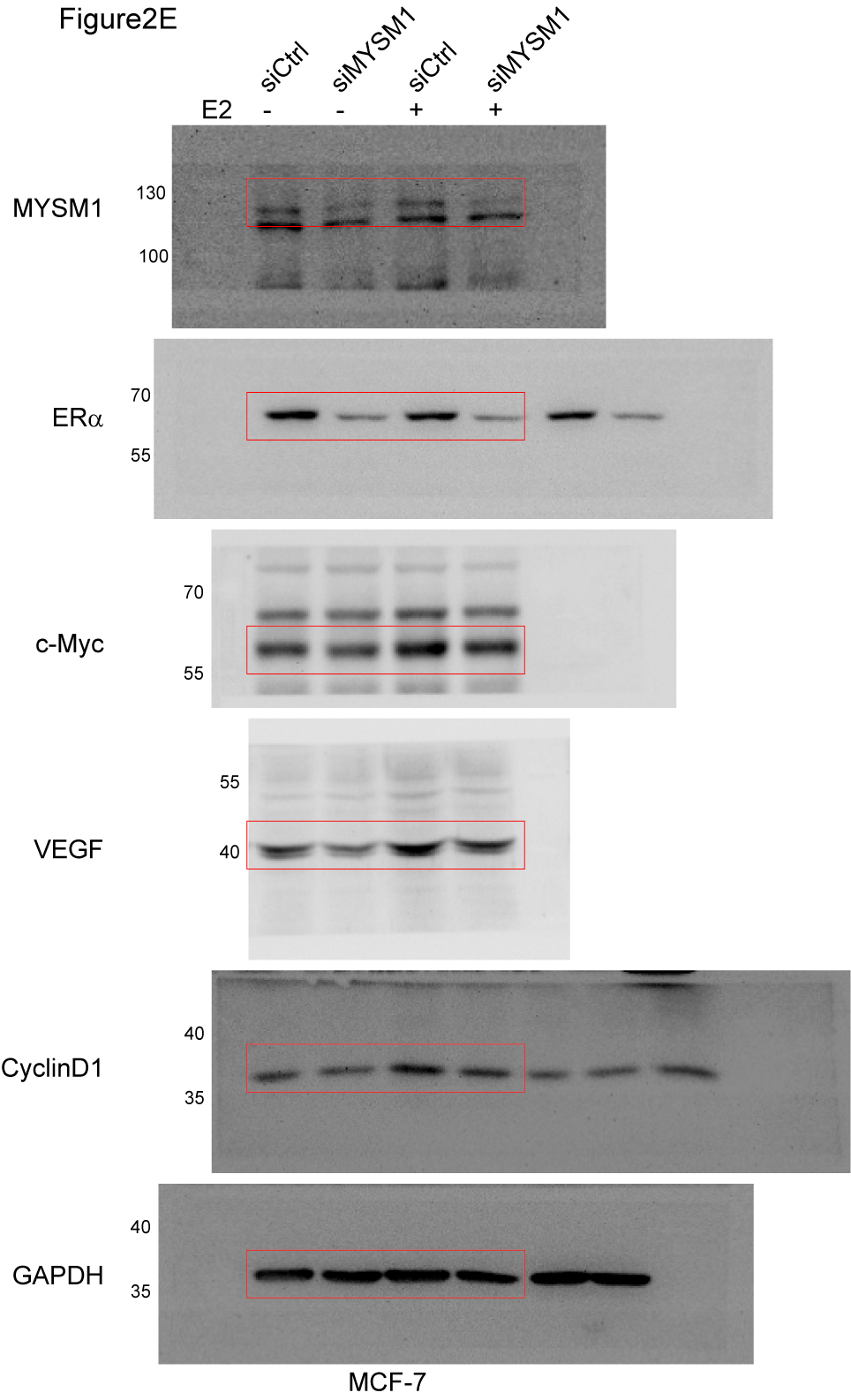

Supplement: Supplementary file 7 — Source Data Fig. 2 [file 44321_2023_3_MOESM7_ESM.zip › Figure 2/Fig 2E-Image data.tif]

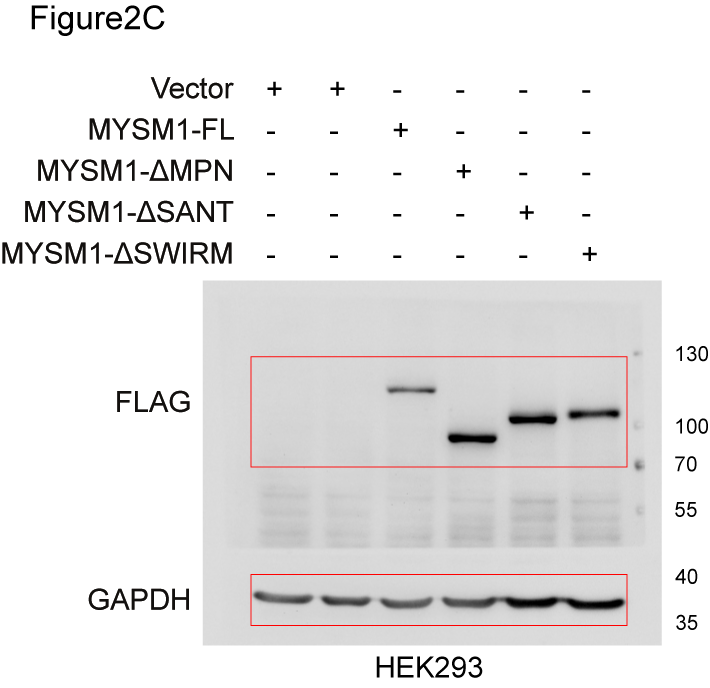

Supplement: Supplementary file 7 — Source Data Fig. 2 [file 44321_2023_3_MOESM7_ESM.zip › Figure 2/Fig 2C-Image data.tif]

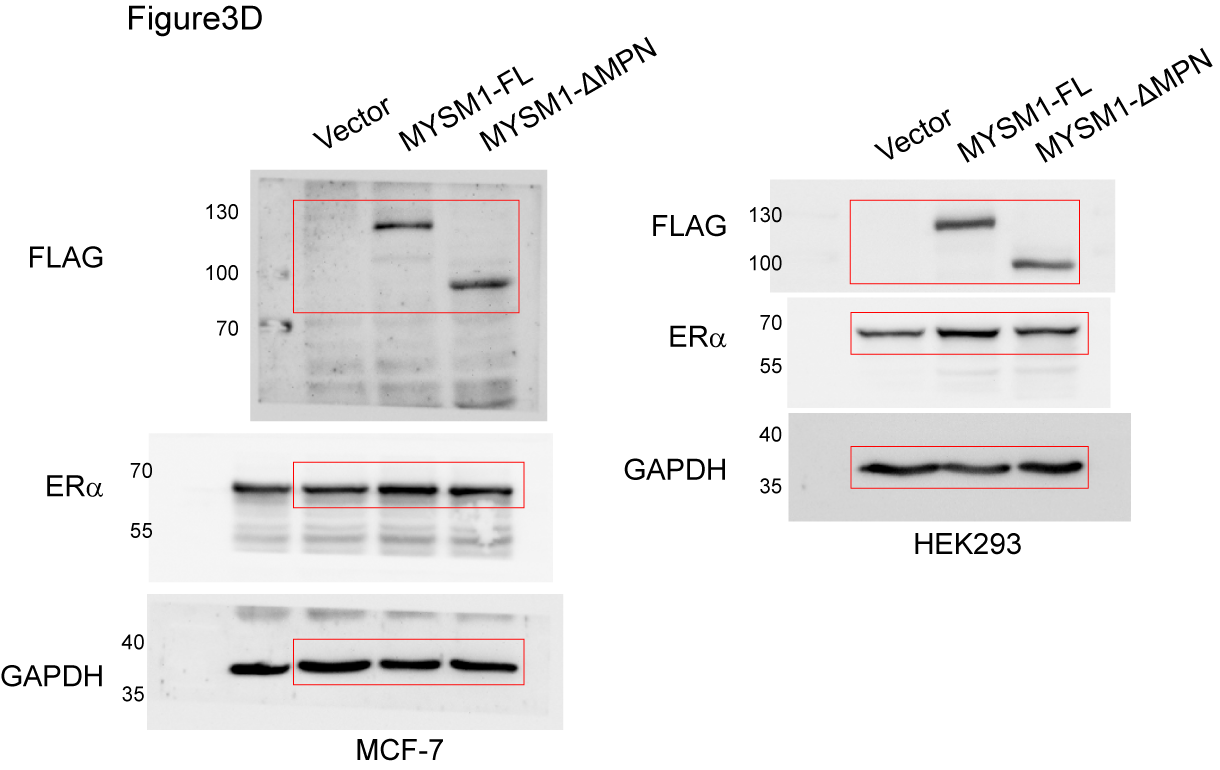

Supplement: Supplementary file 8 — Source Data Fig. 3 [file 44321_2023_3_MOESM8_ESM.zip › Figure 3/Fig 3D-Image data.tif]

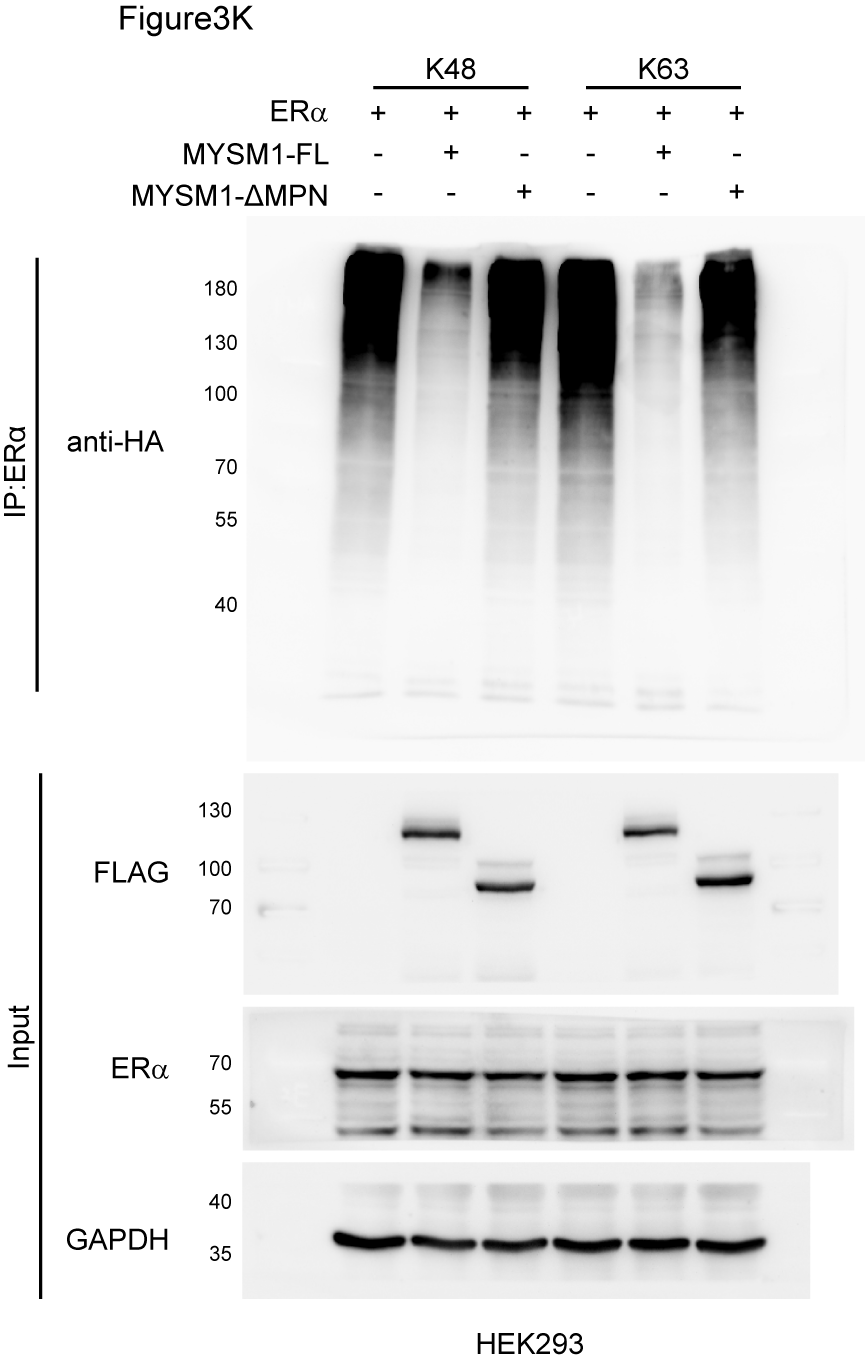

Supplement: Supplementary file 8 — Source Data Fig. 3 [file 44321_2023_3_MOESM8_ESM.zip › Figure 3/Fig 3K-Image data.tif]

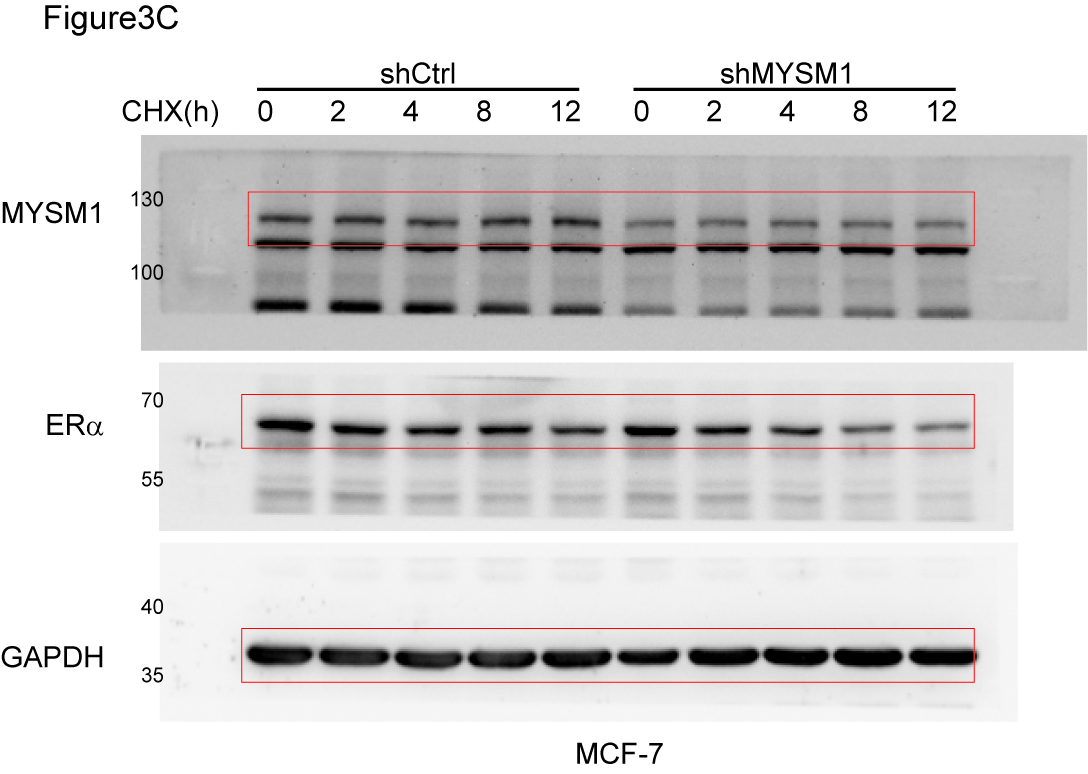

Supplement: Supplementary file 8 — Source Data Fig. 3 [file 44321_2023_3_MOESM8_ESM.zip › Figure 3/Fig 3C-Image data.tif]

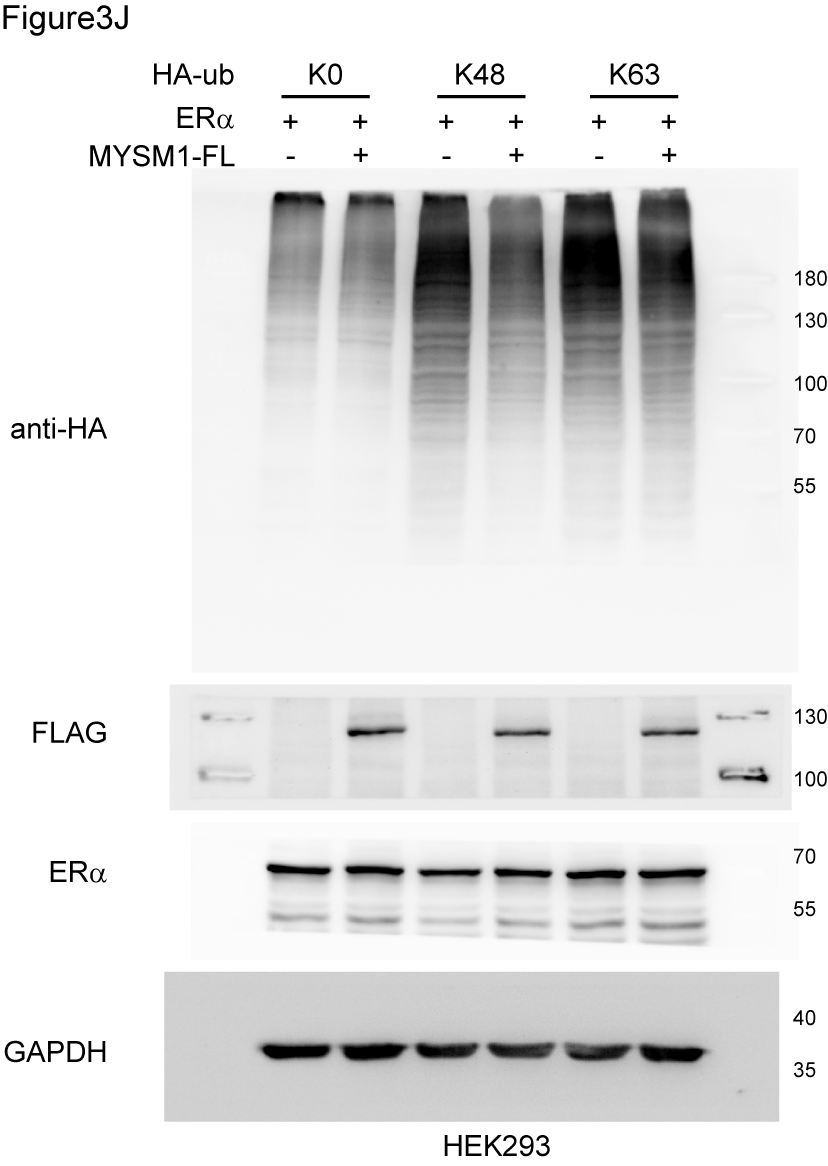

Supplement: Supplementary file 8 — Source Data Fig. 3 [file 44321_2023_3_MOESM8_ESM.zip › Figure 3/Fig 3J-Image data.tif]

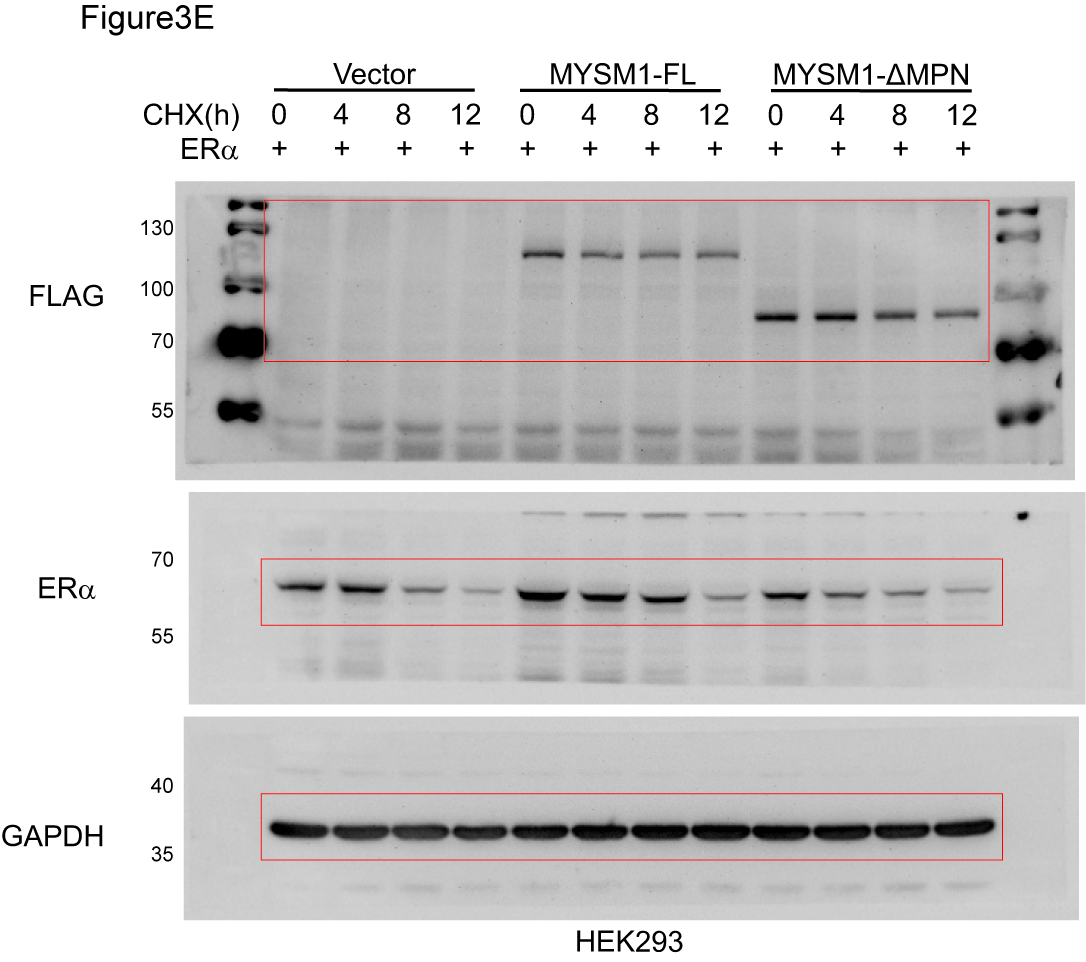

Supplement: Supplementary file 8 — Source Data Fig. 3 [file 44321_2023_3_MOESM8_ESM.zip › Figure 3/Fig 3E-Image data.tif]

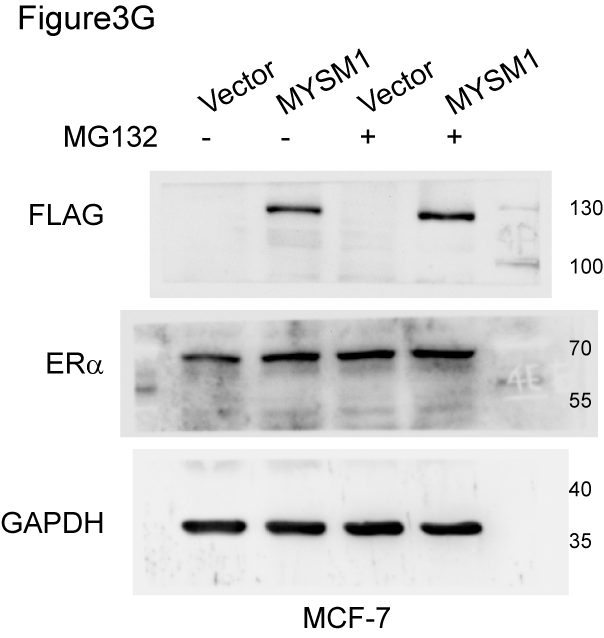

Supplement: Supplementary file 8 — Source Data Fig. 3 [file 44321_2023_3_MOESM8_ESM.zip › Figure 3/Fig 3G-Image data.tif]

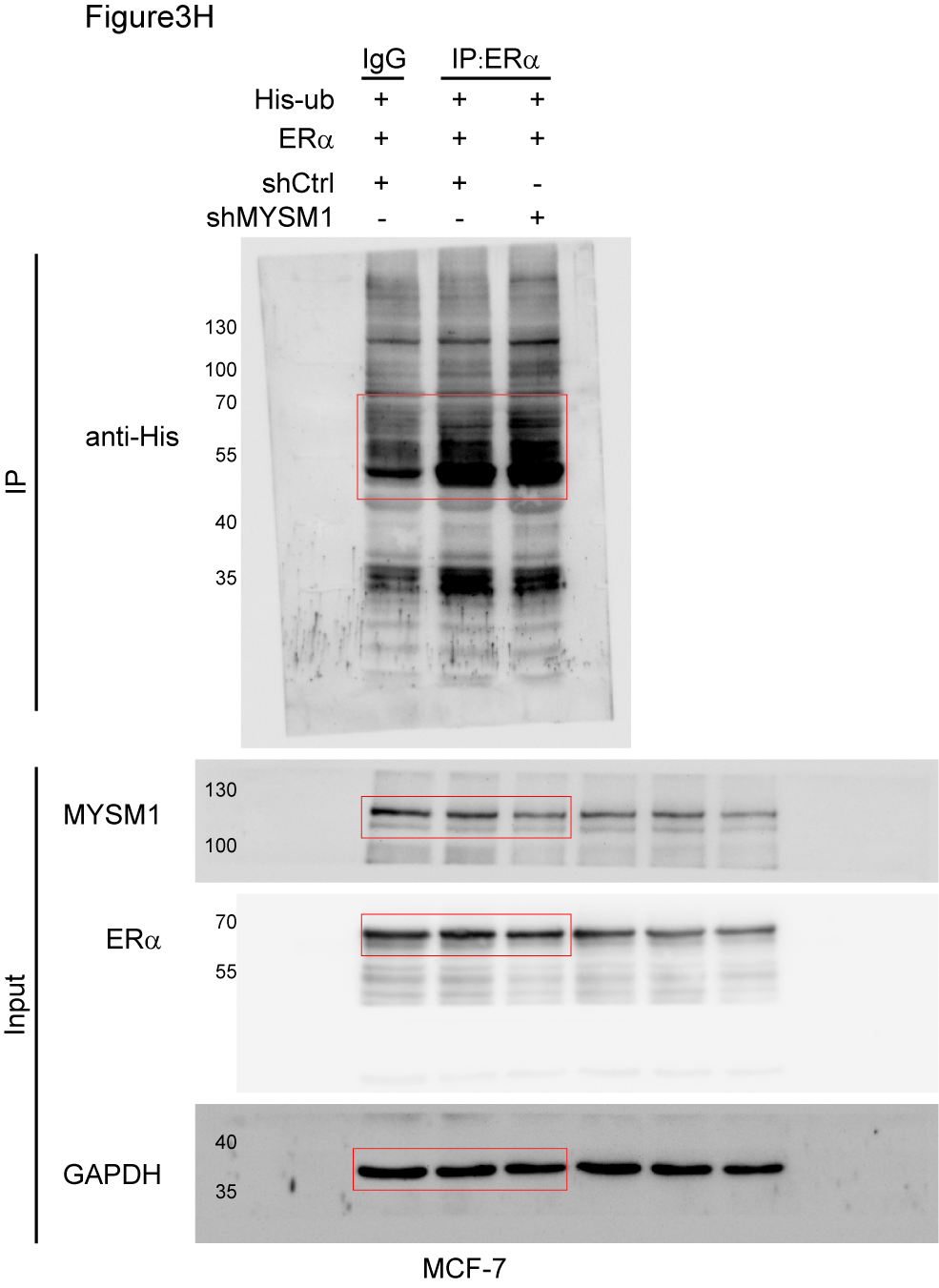

Supplement: Supplementary file 8 — Source Data Fig. 3 [file 44321_2023_3_MOESM8_ESM.zip › Figure 3/Fig 3H-Image data.tif]

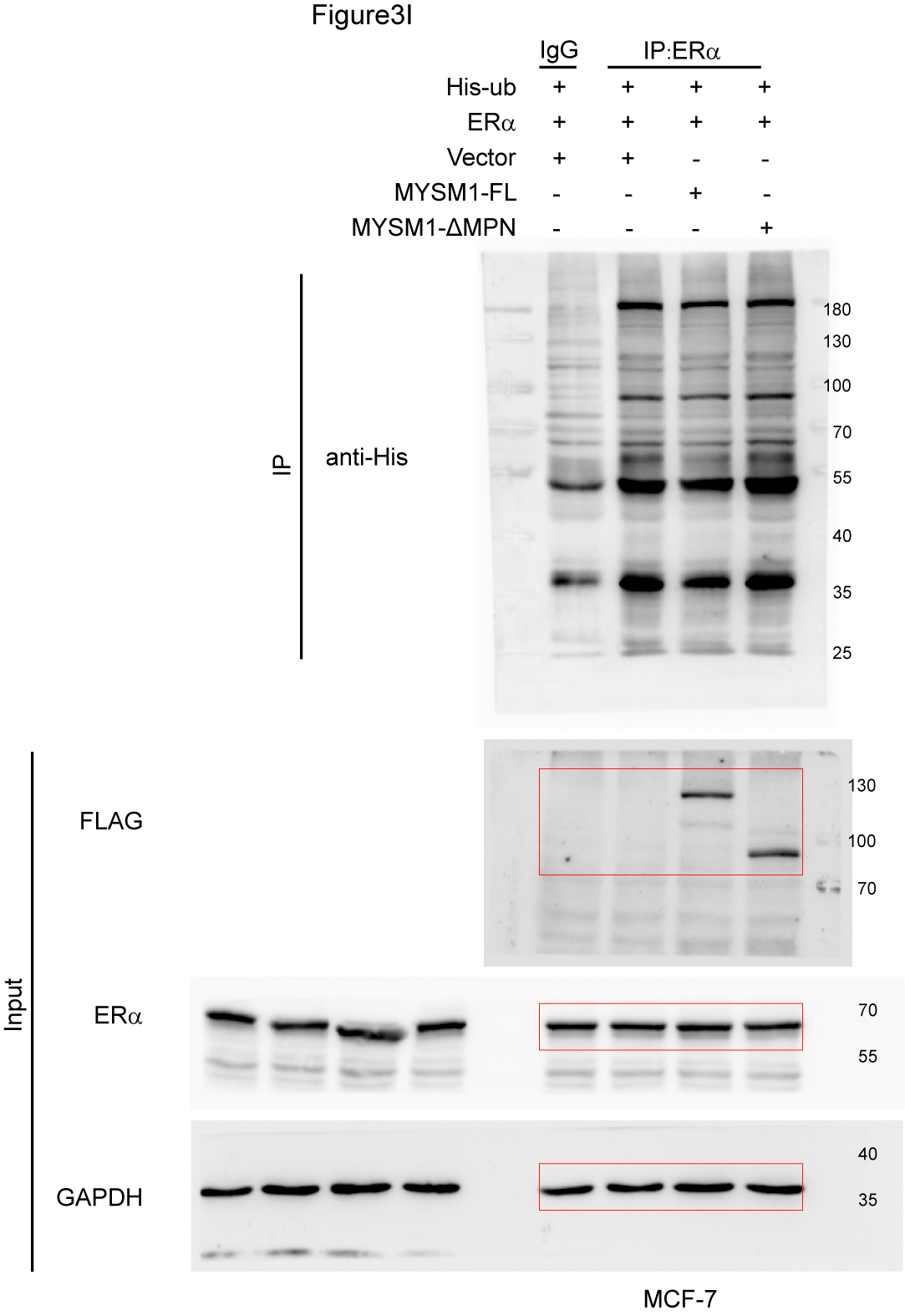

Supplement: Supplementary file 8 — Source Data Fig. 3 [file 44321_2023_3_MOESM8_ESM.zip › Figure 3/Fig 3I-Image data.tif]

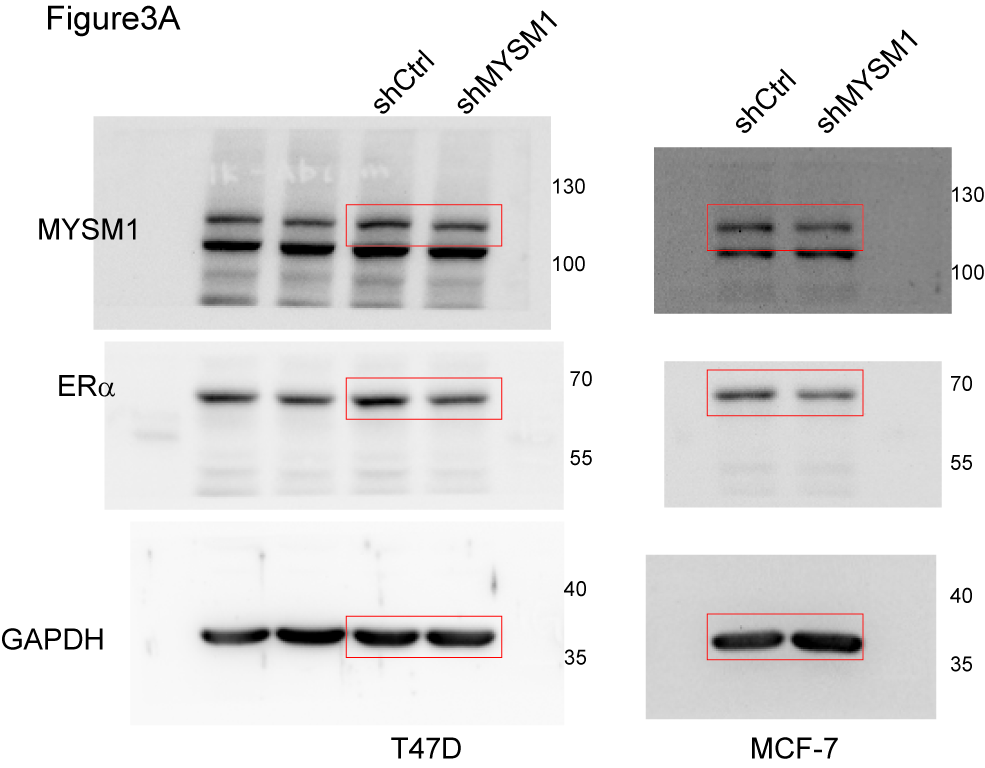

Supplement: Supplementary file 8 — Source Data Fig. 3 [file 44321_2023_3_MOESM8_ESM.zip › Figure 3/Fig 3A-Image data.tif]

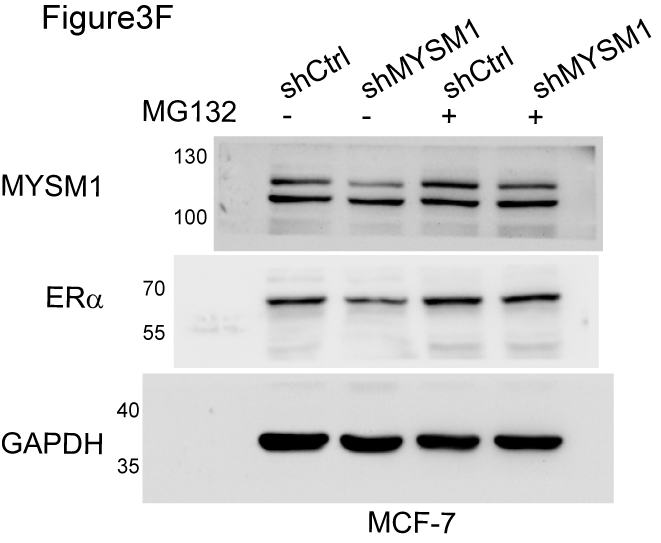

Supplement: Supplementary file 8 — Source Data Fig. 3 [file 44321_2023_3_MOESM8_ESM.zip › Figure 3/Fig 3F-Image data.tif]

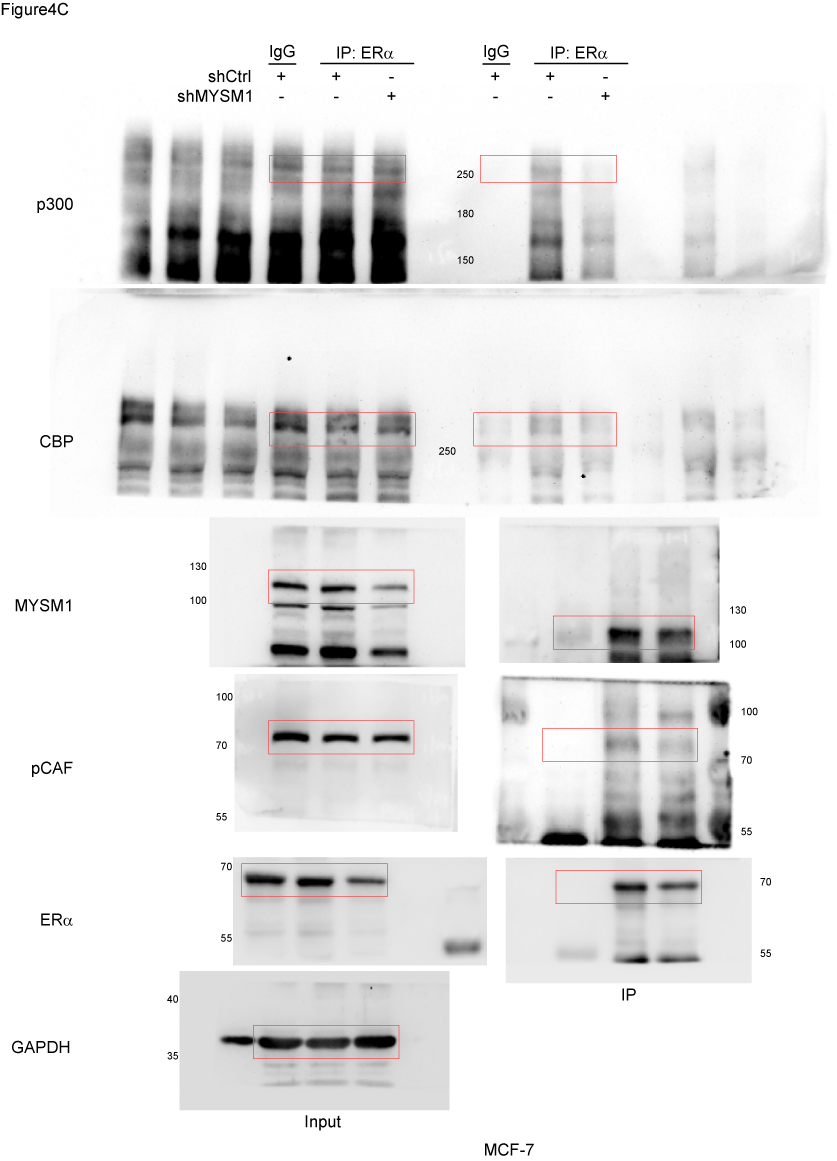

Supplement: Supplementary file 9 — Source Data Fig. 4 [file 44321_2023_3_MOESM9_ESM.zip › Figure 4/Fig 4C-Image data.tif]

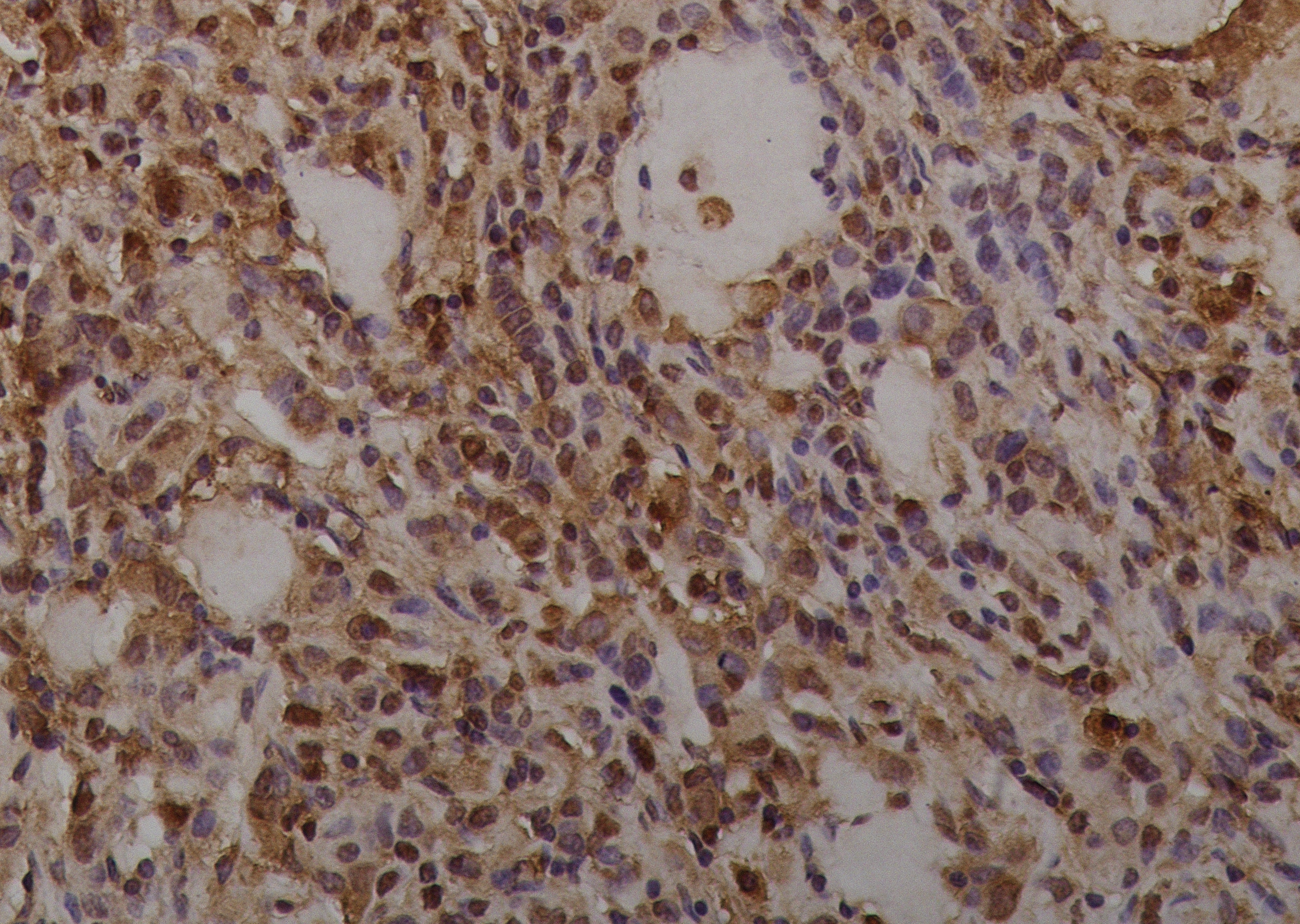

Supplement: Supplementary file 10 — Source Data Fig. 5 [file 44321_2023_3_MOESM10_ESM.zip › Figure 5/Fig 5H-Image data shCtrl anti-MYSM1.tif]

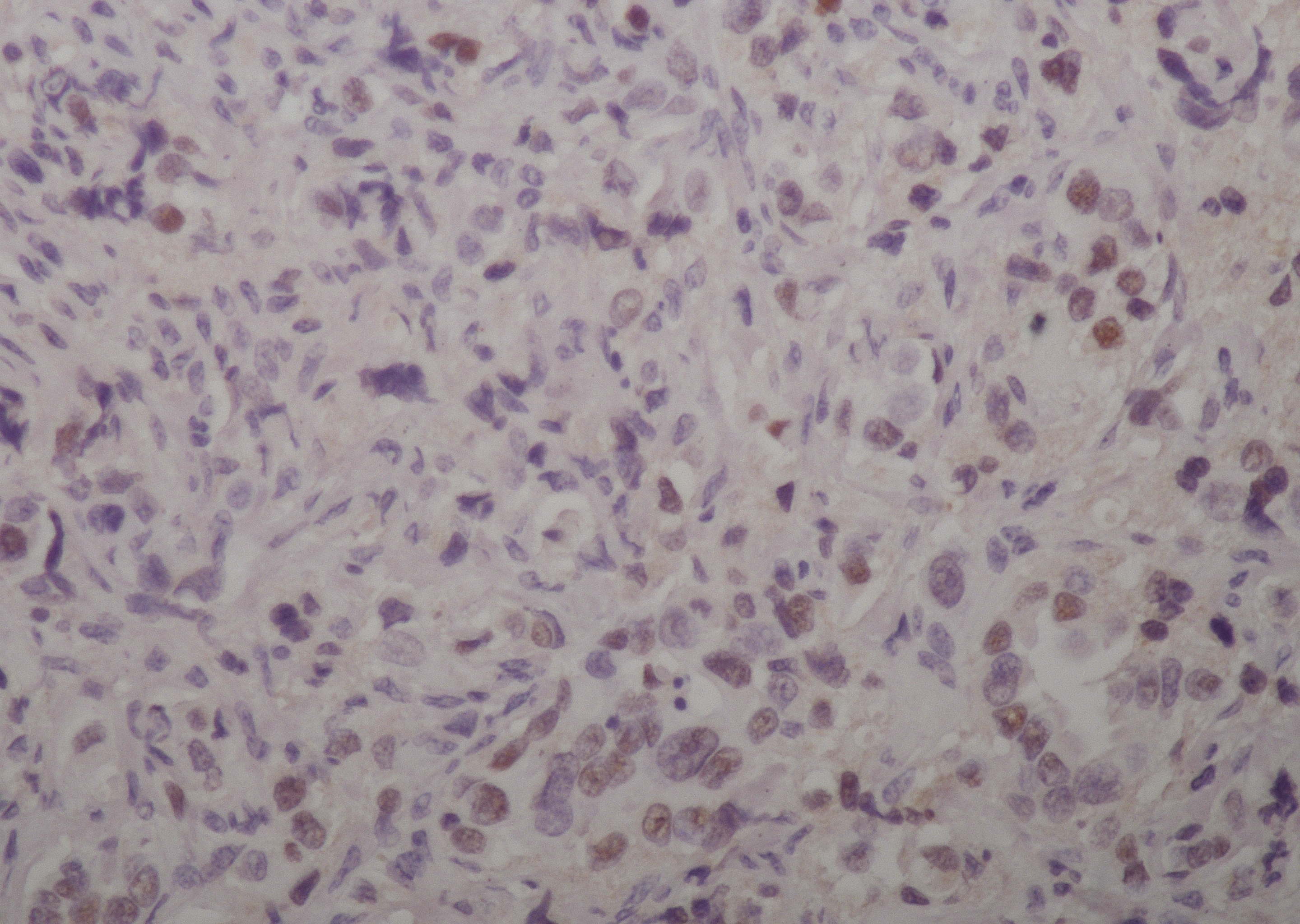

Supplement: Supplementary file 10 — Source Data Fig. 5 [file 44321_2023_3_MOESM10_ESM.zip › Figure 5/Fig 5H-Image data shMYSM1 anti-ERa.tif]

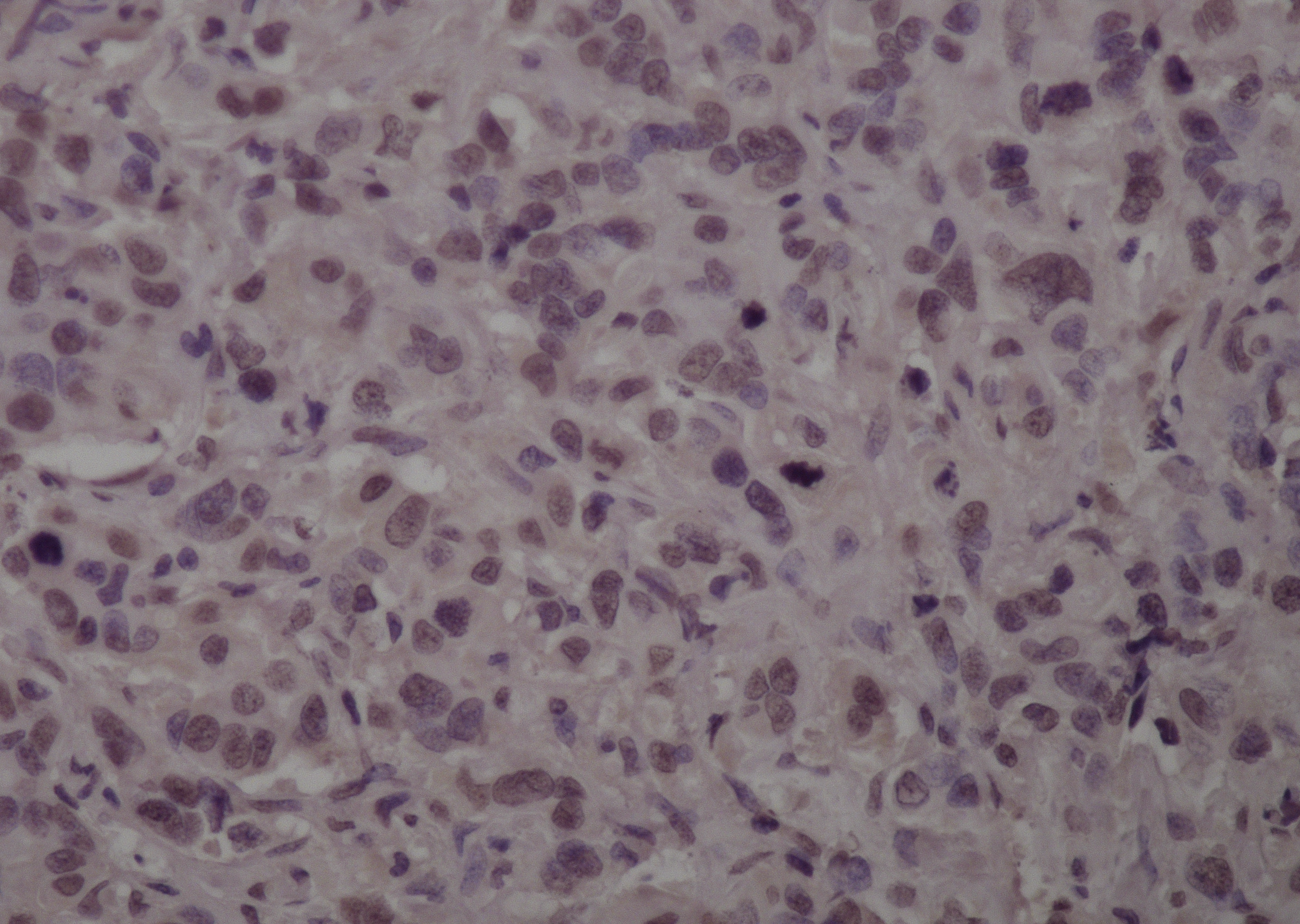

Supplement: Supplementary file 10 — Source Data Fig. 5 [file 44321_2023_3_MOESM10_ESM.zip › Figure 5/Fig 5H-Image data shMYSM1 anti-MYSM1.tif]

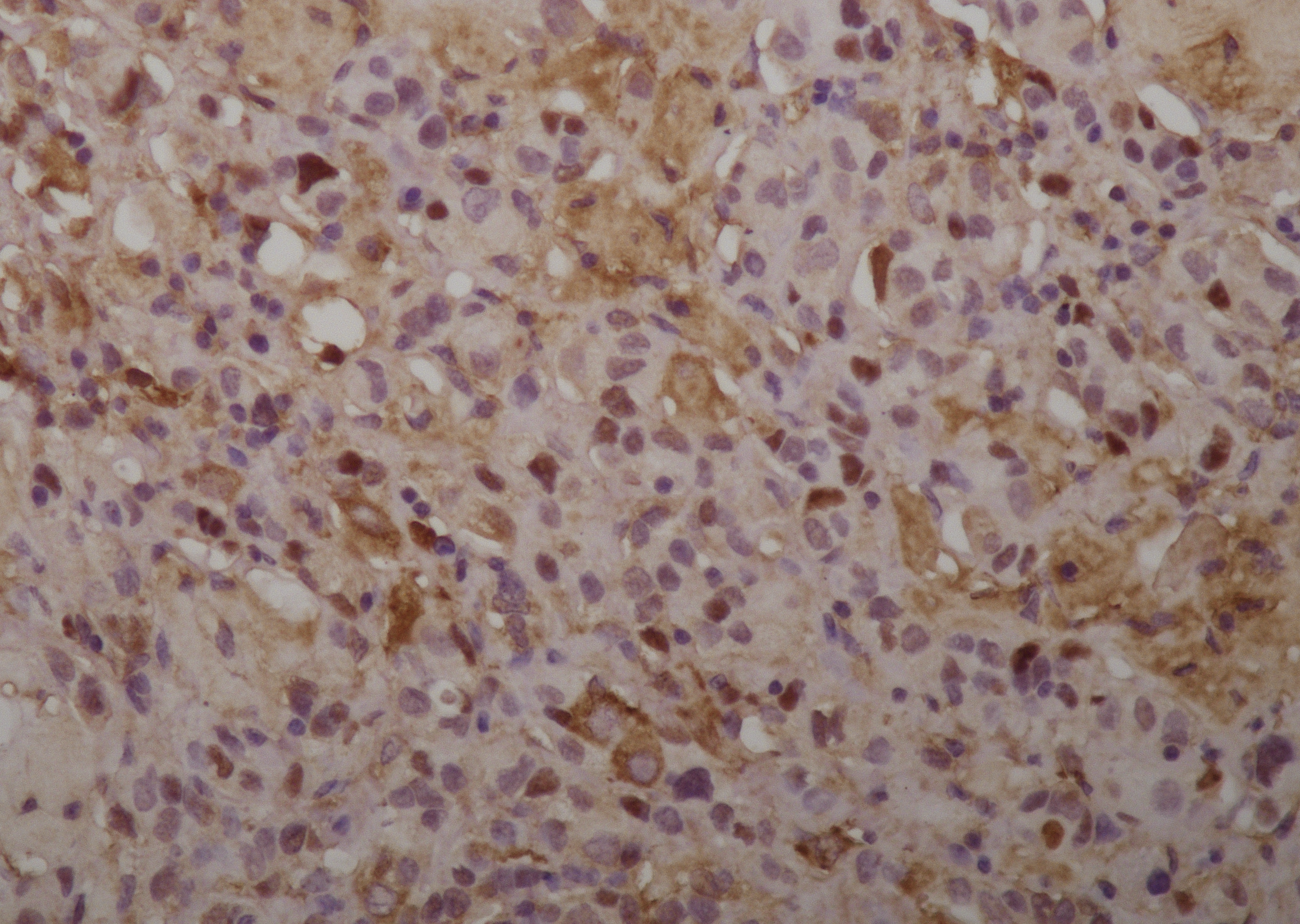

Supplement: Supplementary file 10 — Source Data Fig. 5 [file 44321_2023_3_MOESM10_ESM.zip › Figure 5/Fig 5H-Image data shCtrl anti-ERa.tif]

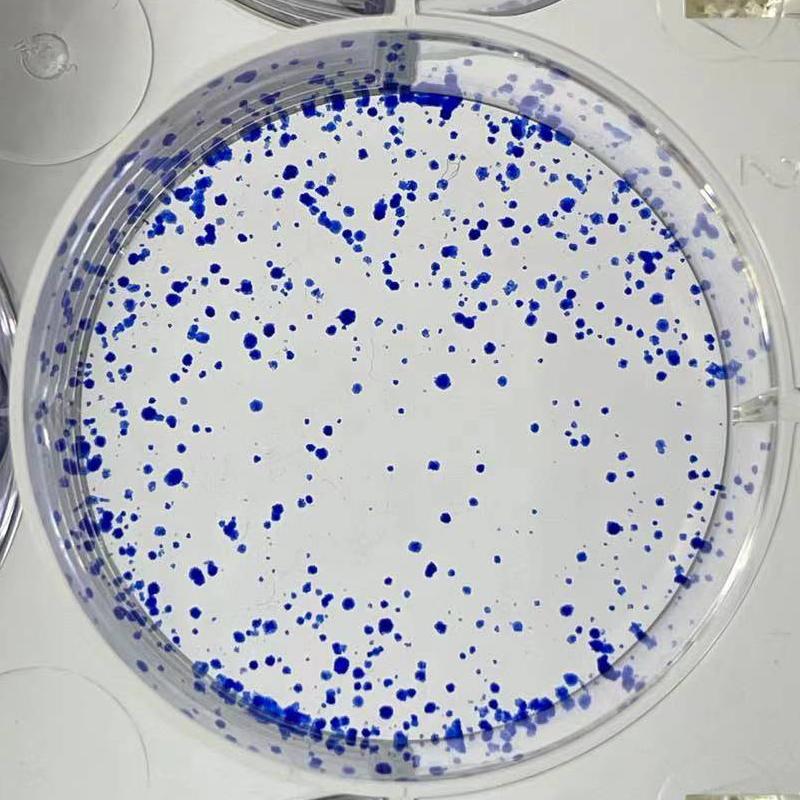

Supplement: Supplementary file 10 — Source Data Fig. 5 [file 44321_2023_3_MOESM10_ESM.zip › Figure 5/Fig 5A-Image data shCtrl E2-.tif]

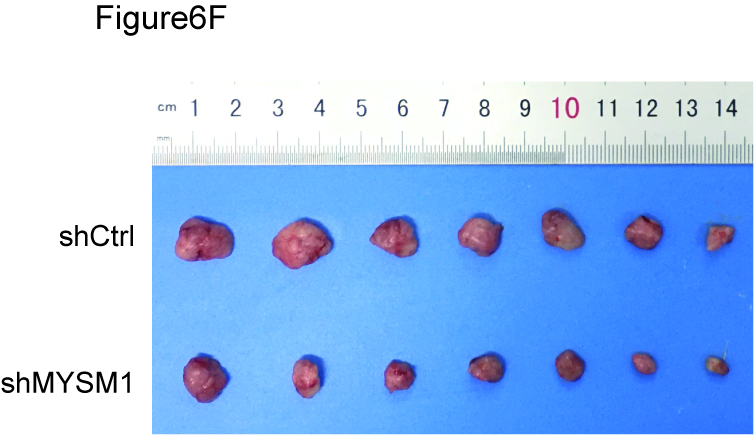

Supplement: Supplementary file 10 — Source Data Fig. 5 [file 44321_2023_3_MOESM10_ESM.zip › Figure 5/Fig 5F-Image data.tif]

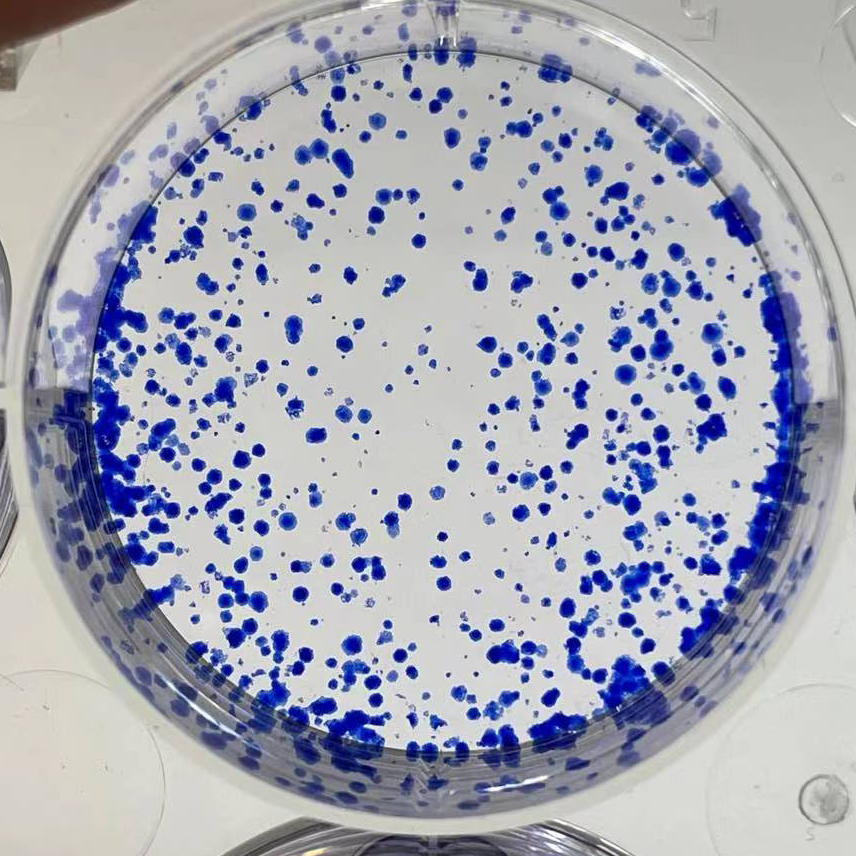

Supplement: Supplementary file 10 — Source Data Fig. 5 [file 44321_2023_3_MOESM10_ESM.zip › Figure 5/Fig 5A-Image data shCtrl E2+.tif]

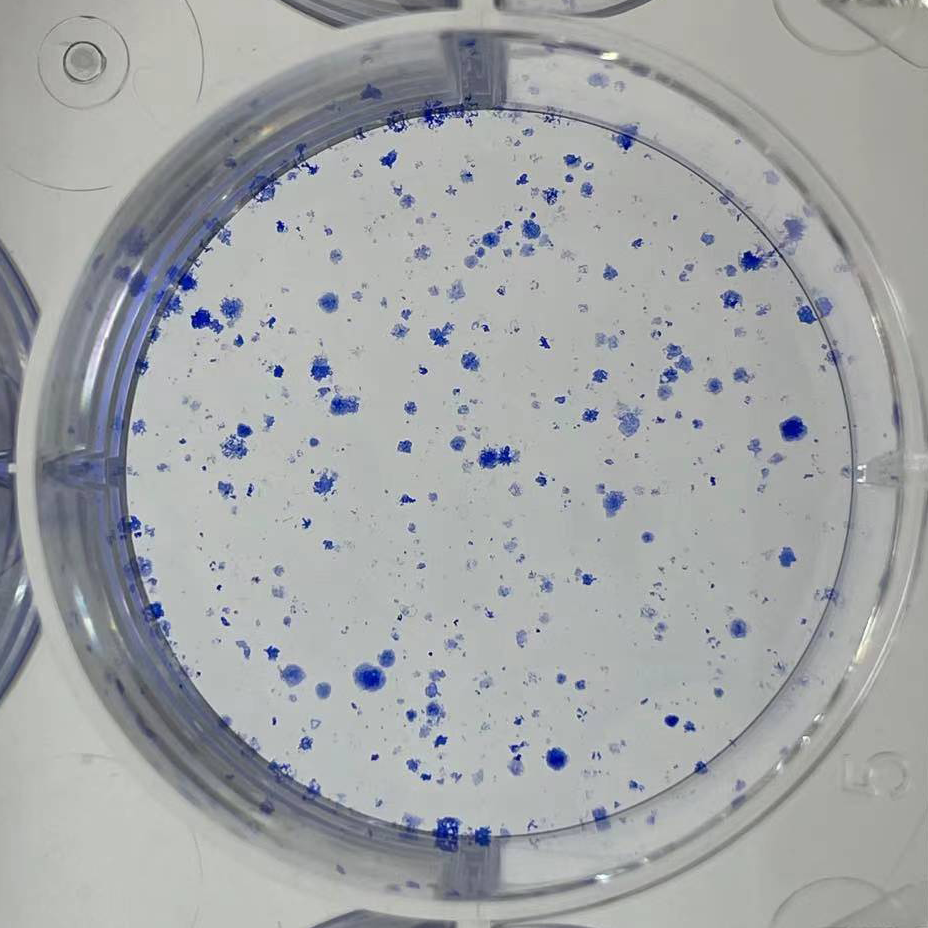

Supplement: Supplementary file 10 — Source Data Fig. 5 [file 44321_2023_3_MOESM10_ESM.zip › Figure 5/Fig 5A-Image data shMYSM1 E2+.tif]

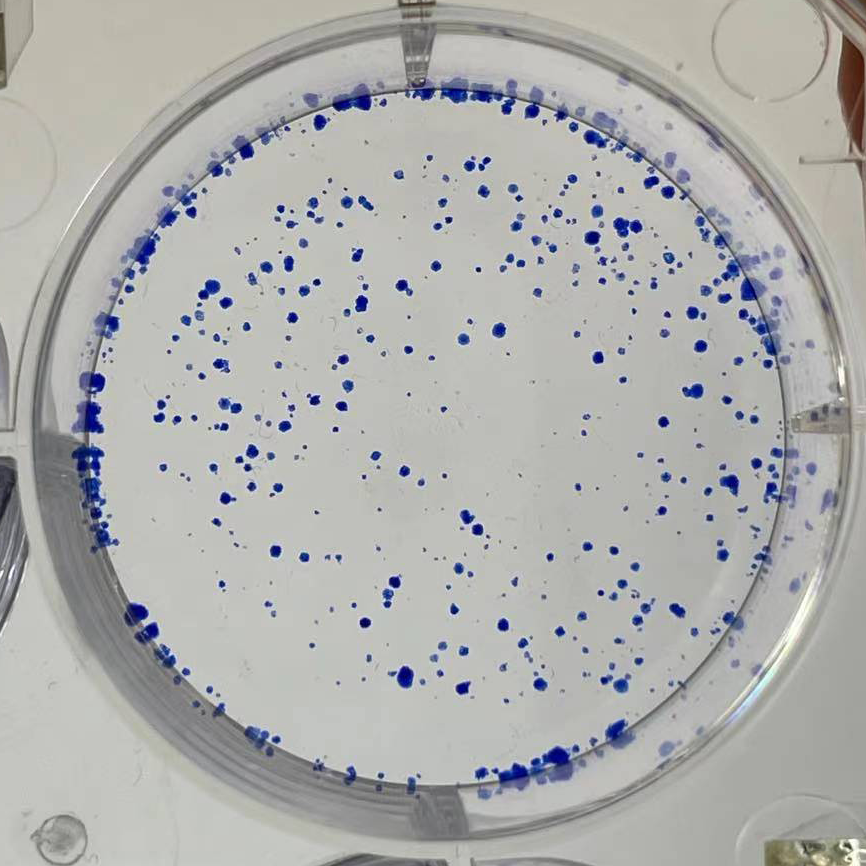

Supplement: Supplementary file 10 — Source Data Fig. 5 [file 44321_2023_3_MOESM10_ESM.zip › Figure 5/Fig 5A-Image data shMYSM1 E2-.tif]

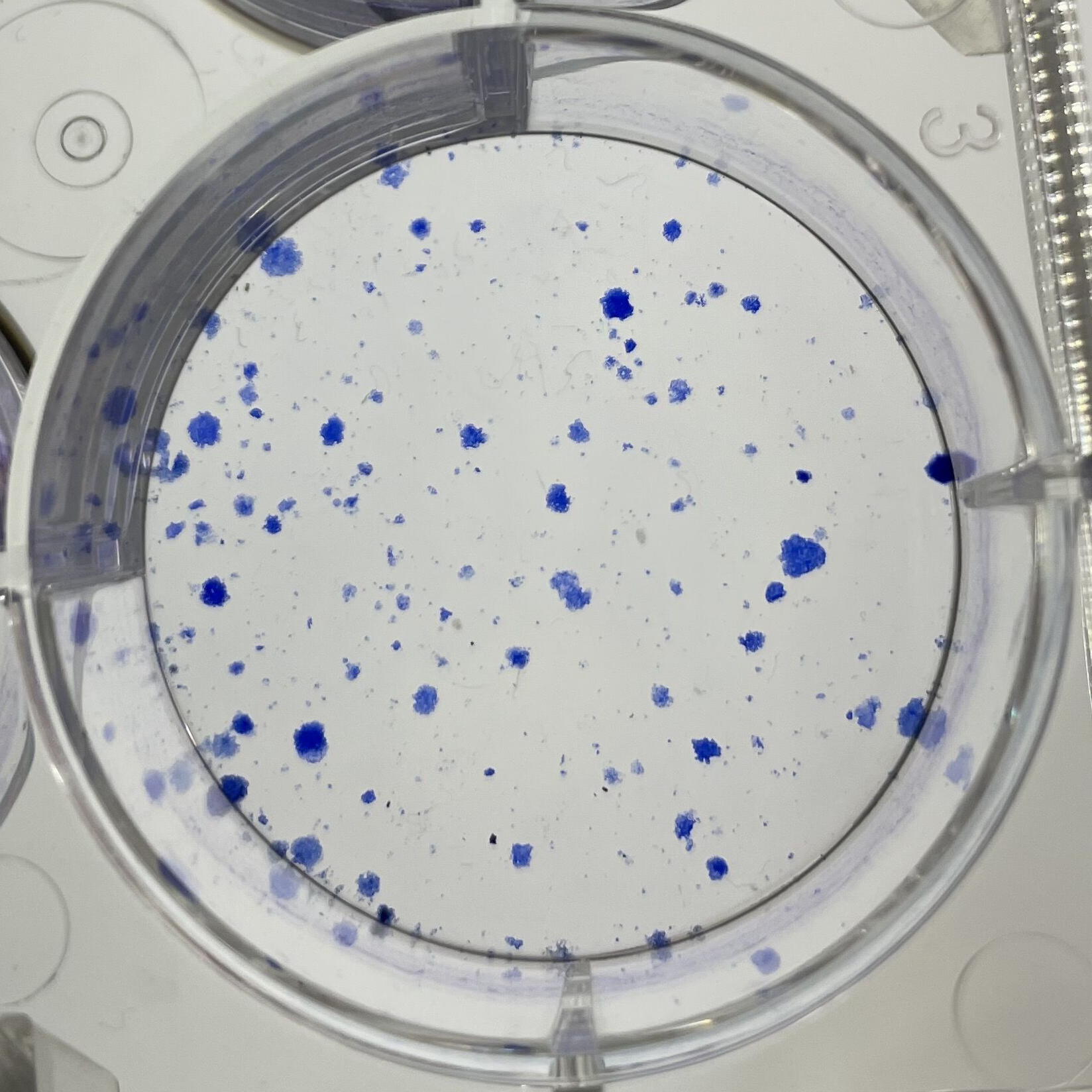

Supplement: Supplementary file 11 — Source Data Fig. 6 [file 44321_2023_3_MOESM11_ESM.zip › Figure 6/Fig 6G-Image data shMYSM1-100nM.tif]

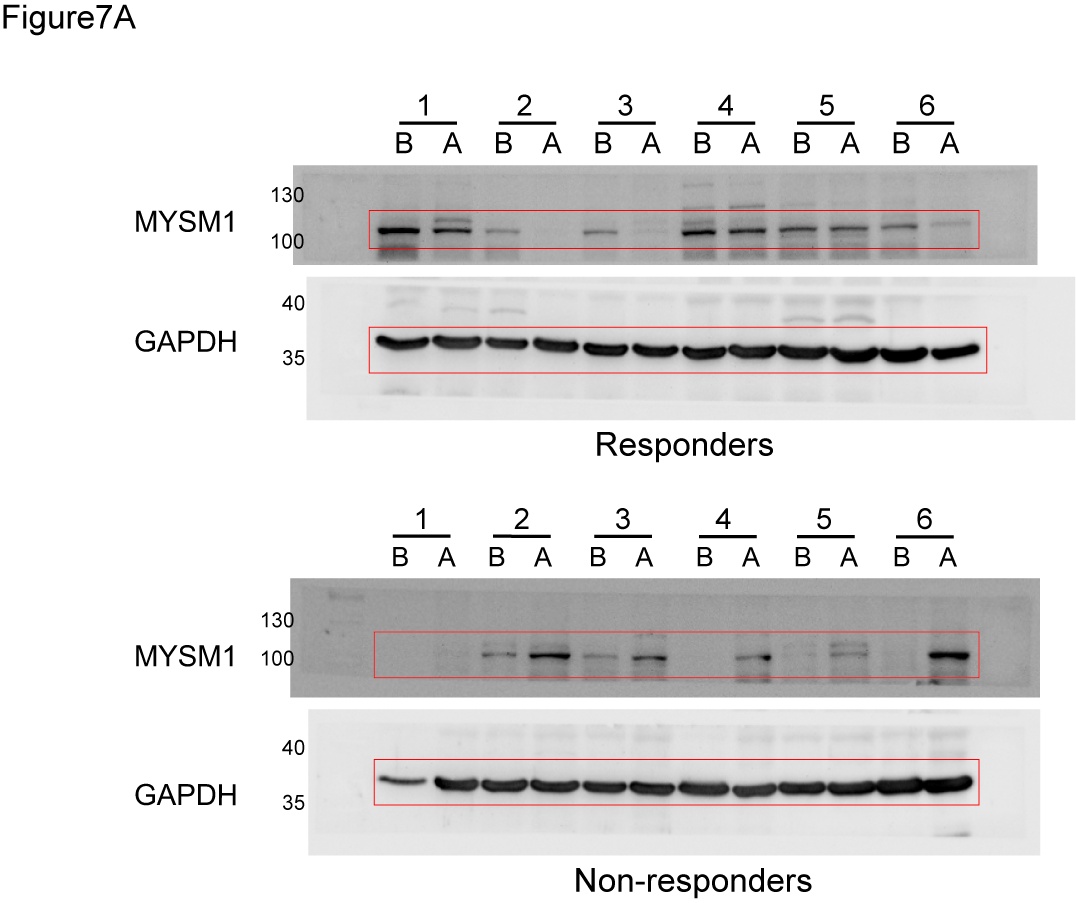

Supplement: Supplementary file 11 — Source Data Fig. 6 [file 44321_2023_3_MOESM11_ESM.zip › Figure 6/Fig 6A-Image data.tif]

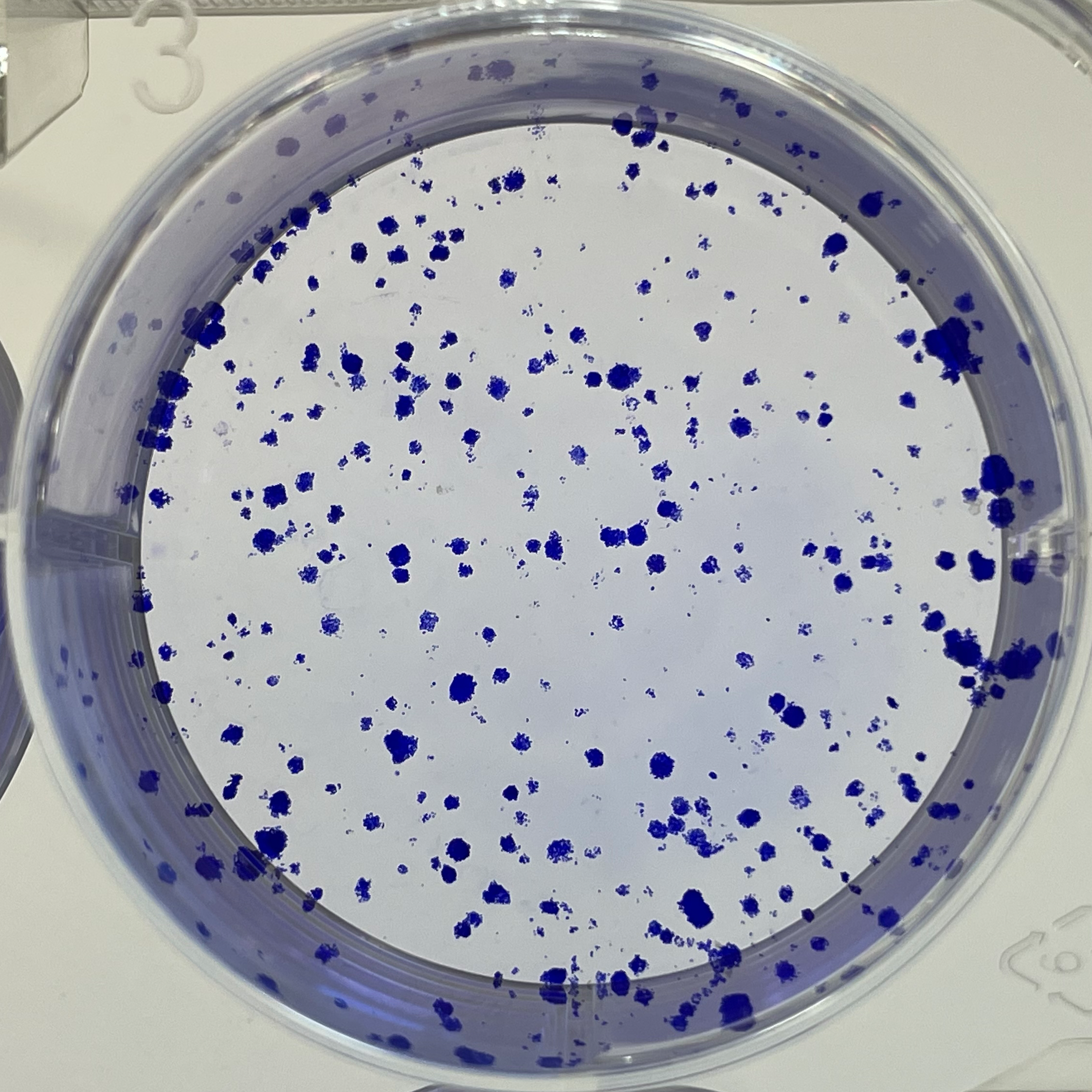

Supplement: Supplementary file 11 — Source Data Fig. 6 [file 44321_2023_3_MOESM11_ESM.zip › Figure 6/Fig 6J-Image data shMYSM1-0nM.tif]

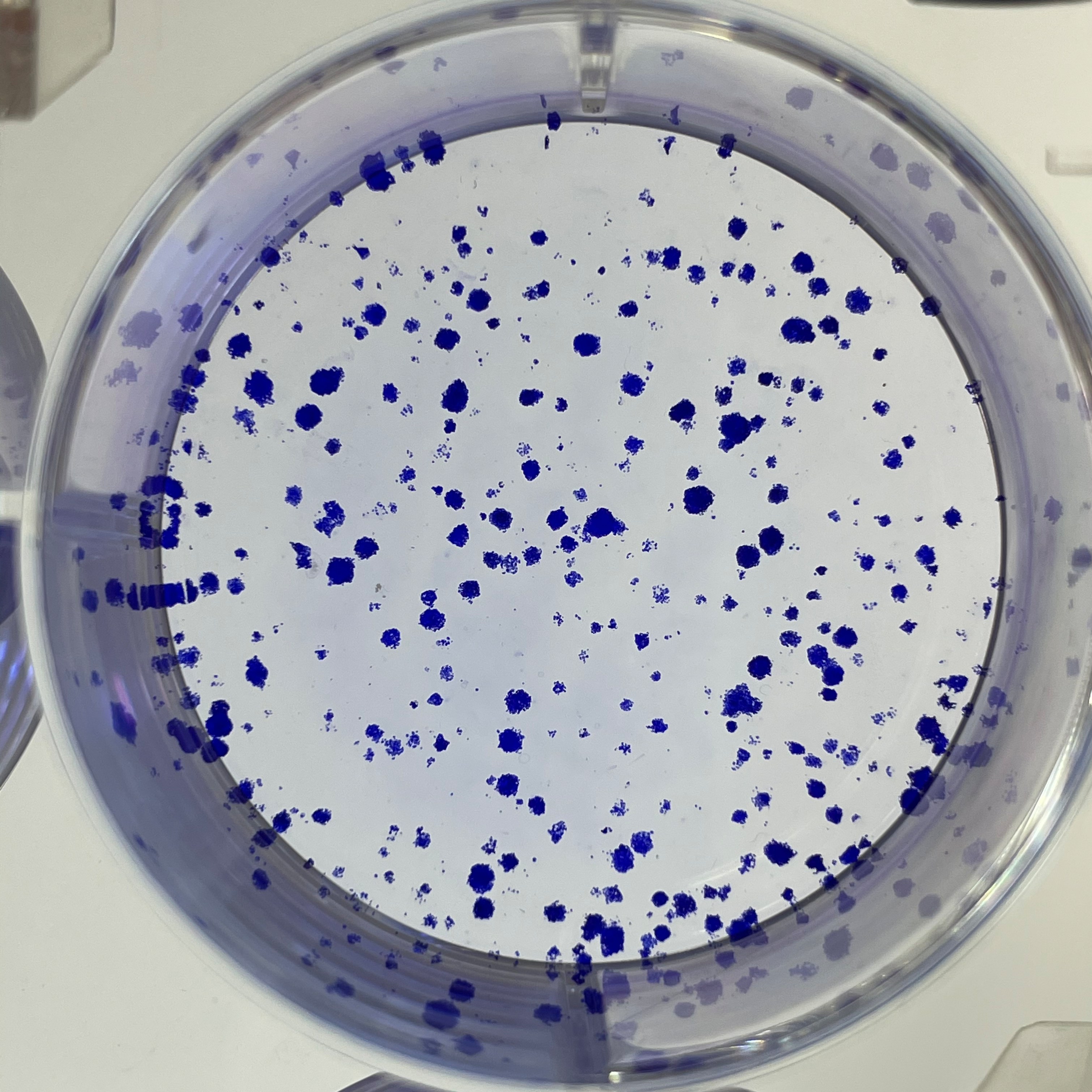

Supplement: Supplementary file 11 — Source Data Fig. 6 [file 44321_2023_3_MOESM11_ESM.zip › Figure 6/Fig 6J-Image data shCtrl-1000nM.tif]

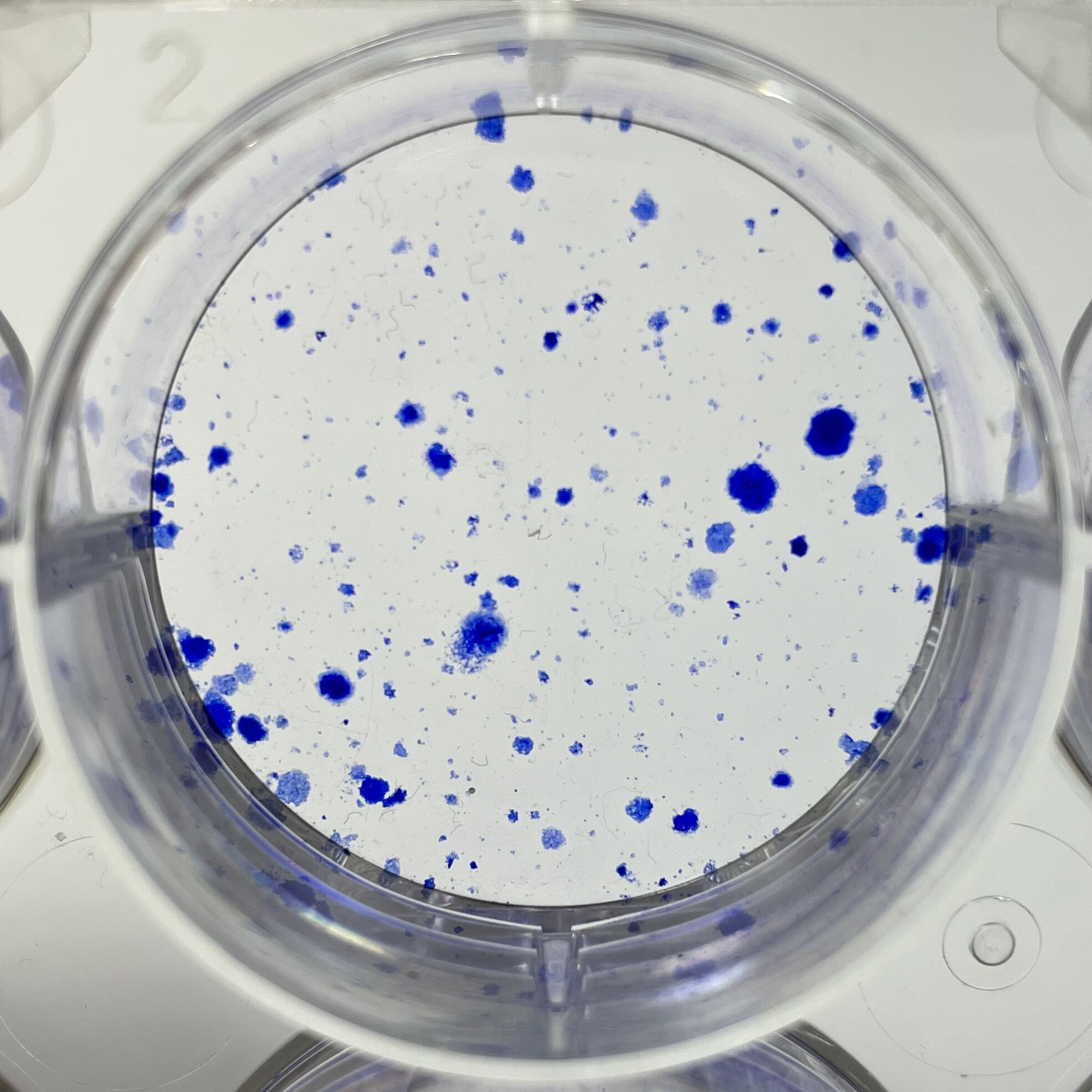

Supplement: Supplementary file 11 — Source Data Fig. 6 [file 44321_2023_3_MOESM11_ESM.zip › Figure 6/Fig 6G-Image data shCtrl-100nM.tif]

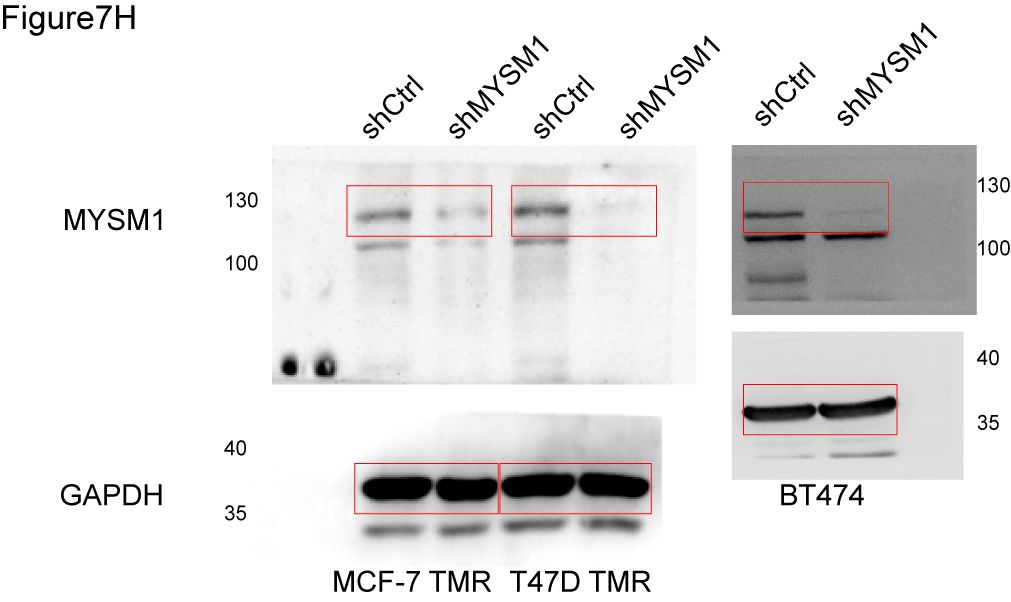

Supplement: Supplementary file 11 — Source Data Fig. 6 [file 44321_2023_3_MOESM11_ESM.zip › Figure 6/Fig 6H-Image data.tif]

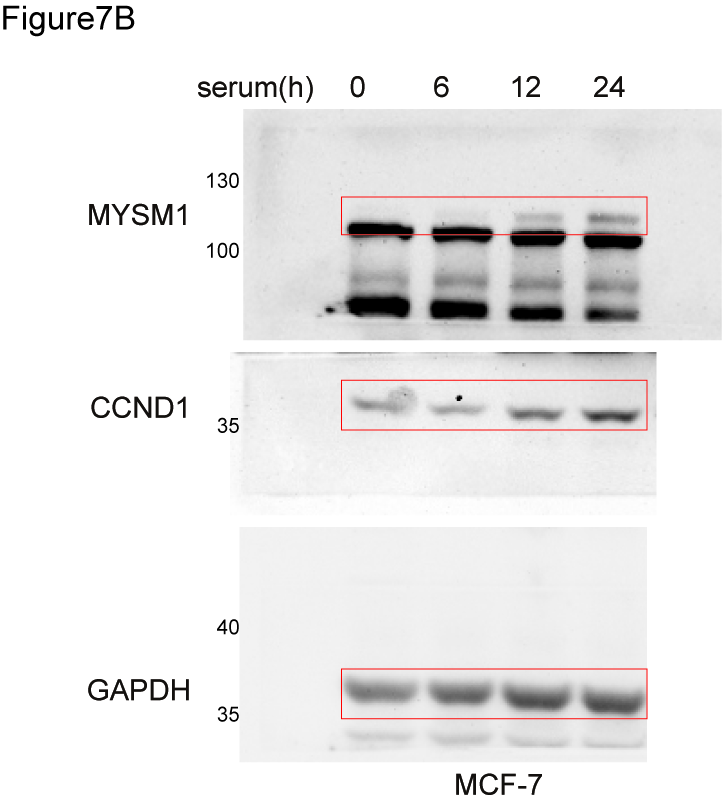

Supplement: Supplementary file 11 — Source Data Fig. 6 [file 44321_2023_3_MOESM11_ESM.zip › Figure 6/Fig 6B-Image data.tif]

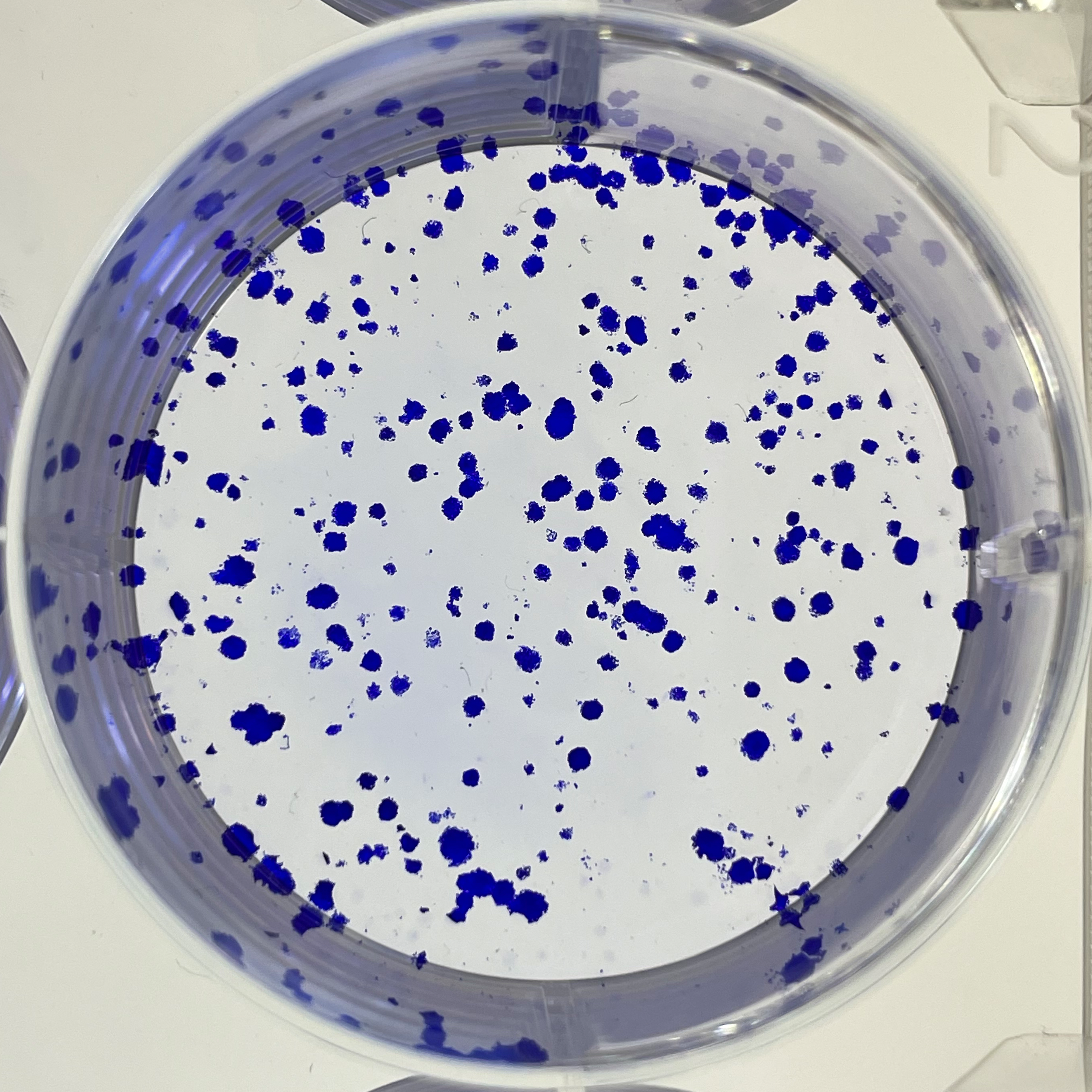

Supplement: Supplementary file 11 — Source Data Fig. 6 [file 44321_2023_3_MOESM11_ESM.zip › Figure 6/Fig 6J-Image data shCtrl-500nM.tif]

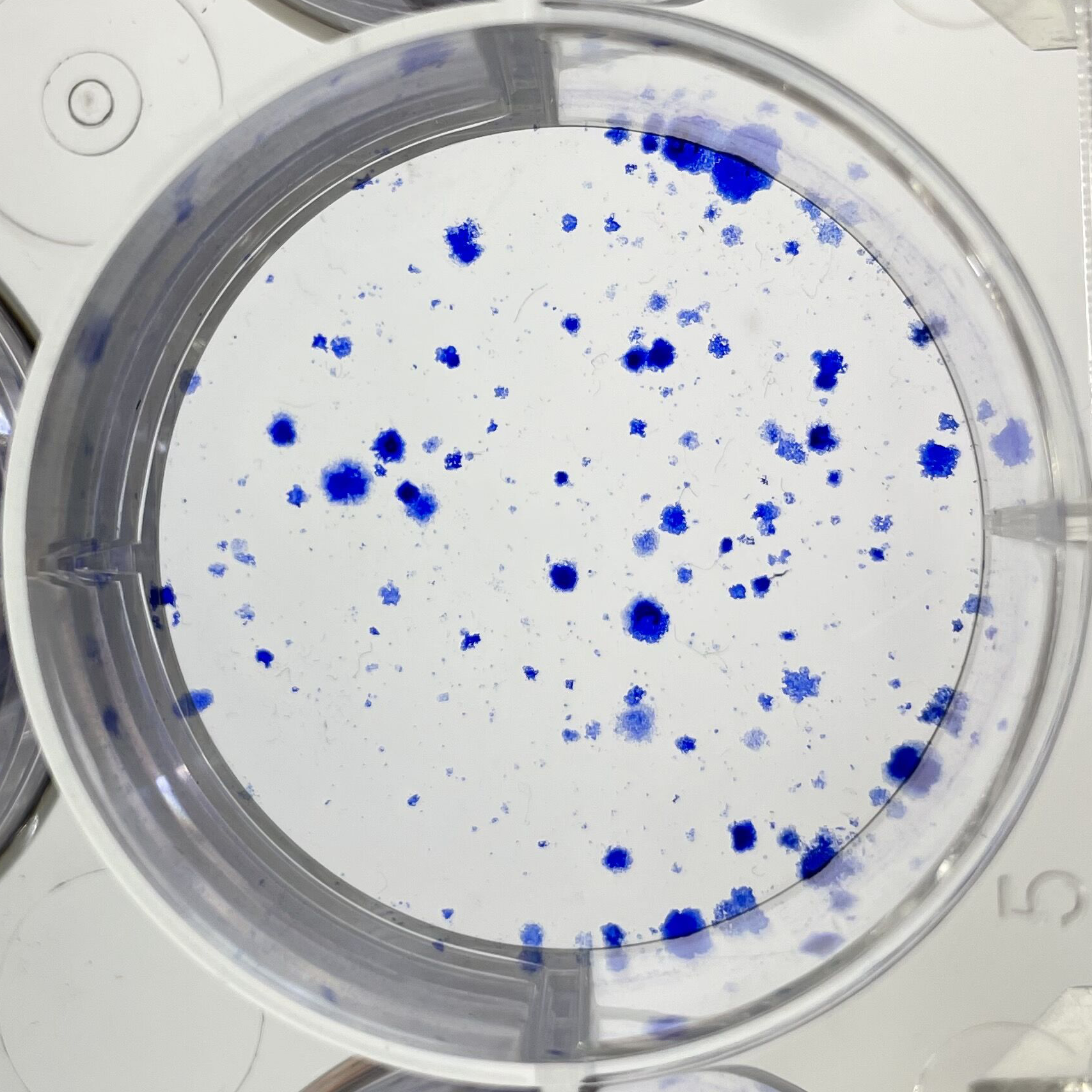

Supplement: Supplementary file 11 — Source Data Fig. 6 [file 44321_2023_3_MOESM11_ESM.zip › Figure 6/Fig 6G-Image data shMYSM1-0nM.tif]

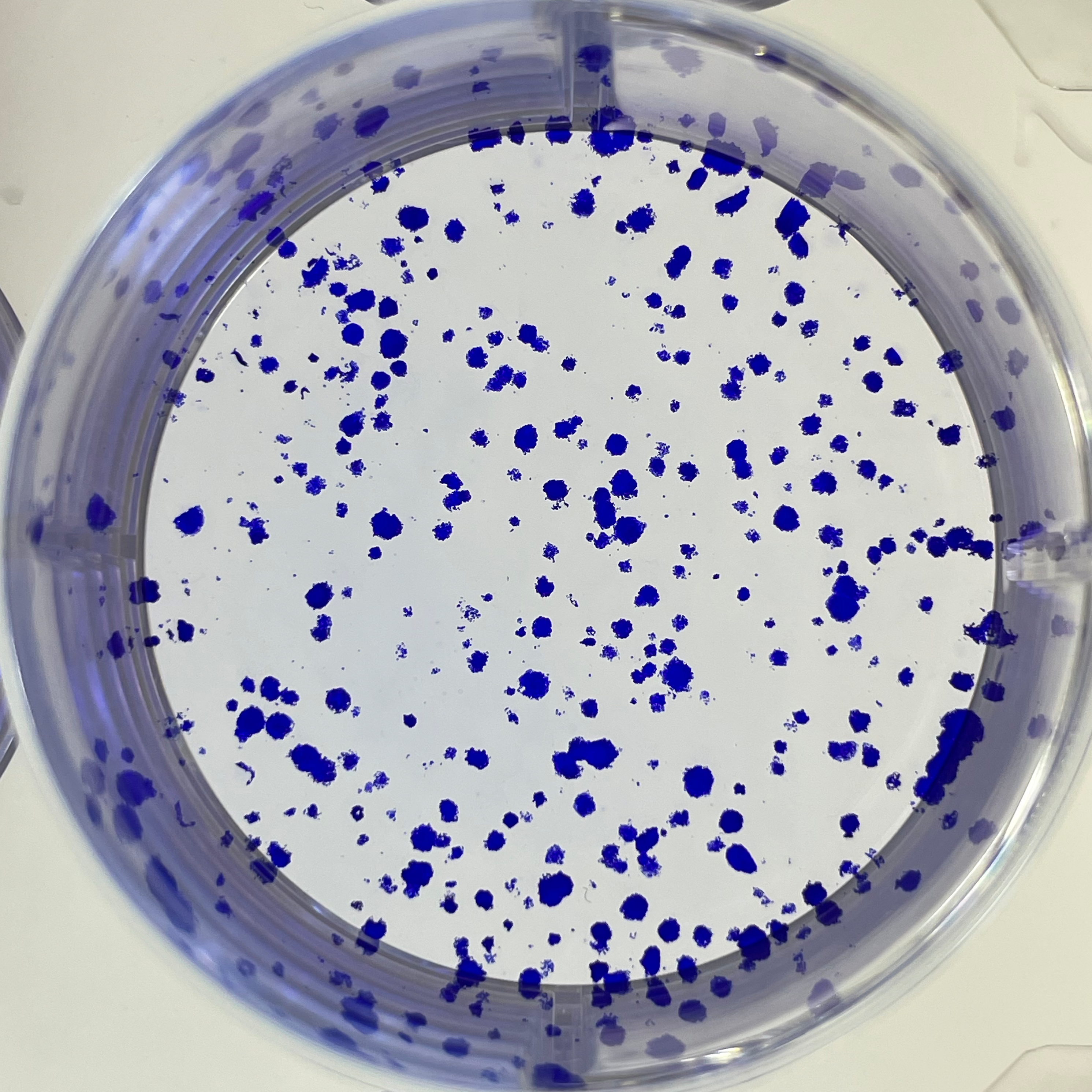

Supplement: Supplementary file 11 — Source Data Fig. 6 [file 44321_2023_3_MOESM11_ESM.zip › Figure 6/Fig 6J-Image data shCtrl-0nM.tif]

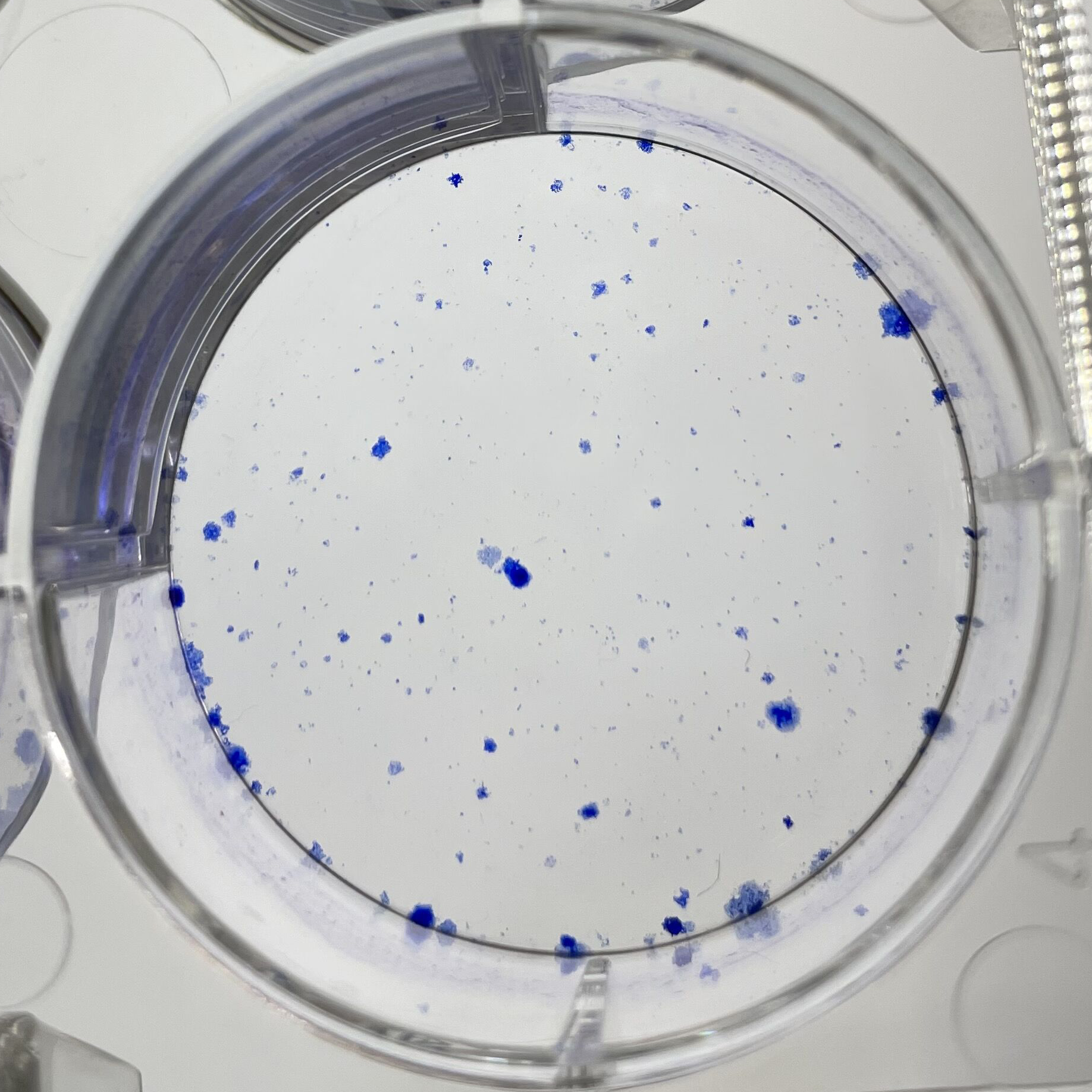

Supplement: Supplementary file 11 — Source Data Fig. 6 [file 44321_2023_3_MOESM11_ESM.zip › Figure 6/Fig 6G-Image data shMYSM1-500nM.tif]

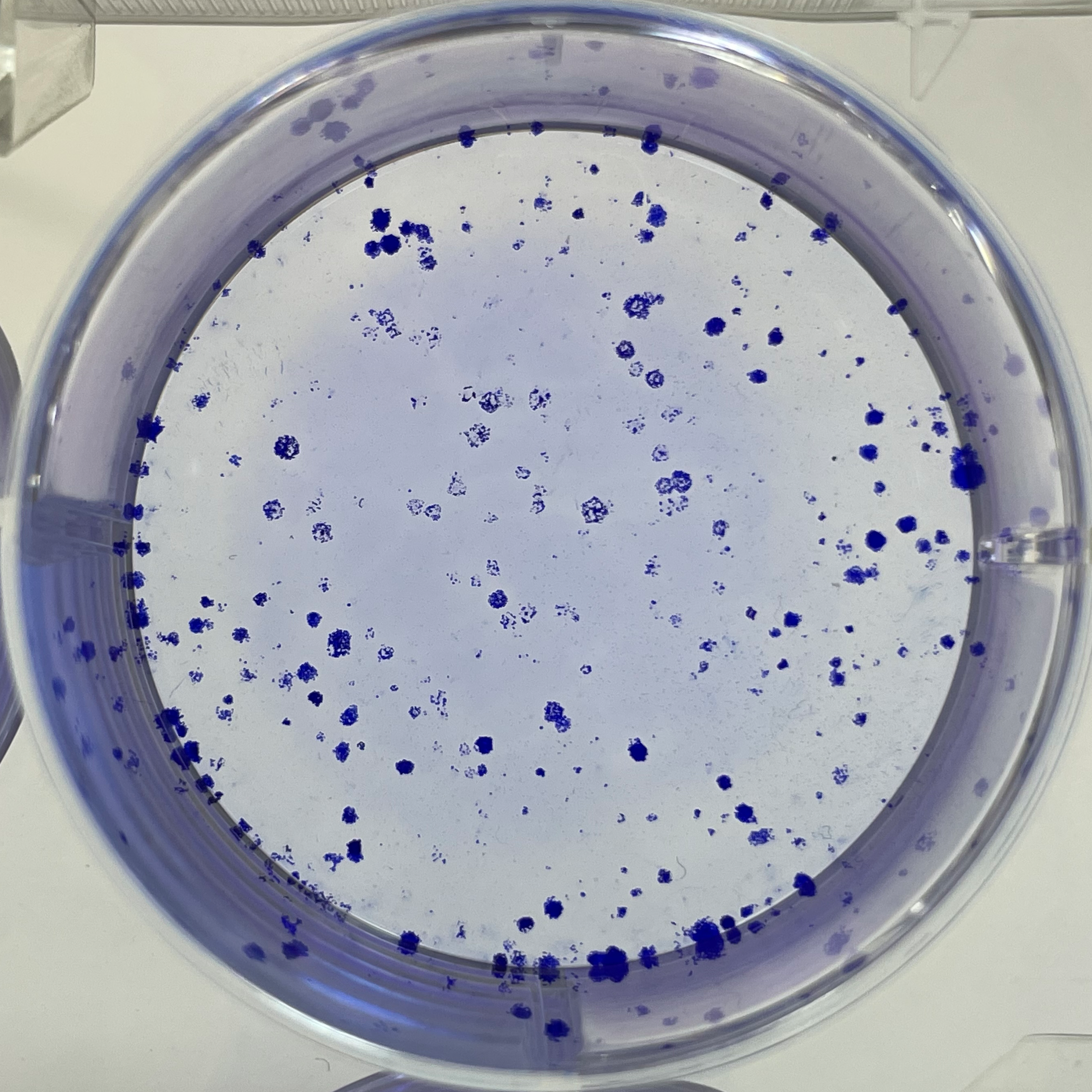

Supplement: Supplementary file 11 — Source Data Fig. 6 [file 44321_2023_3_MOESM11_ESM.zip › Figure 6/Fig 6J-Image data shMYSM1-1000nM.tif]

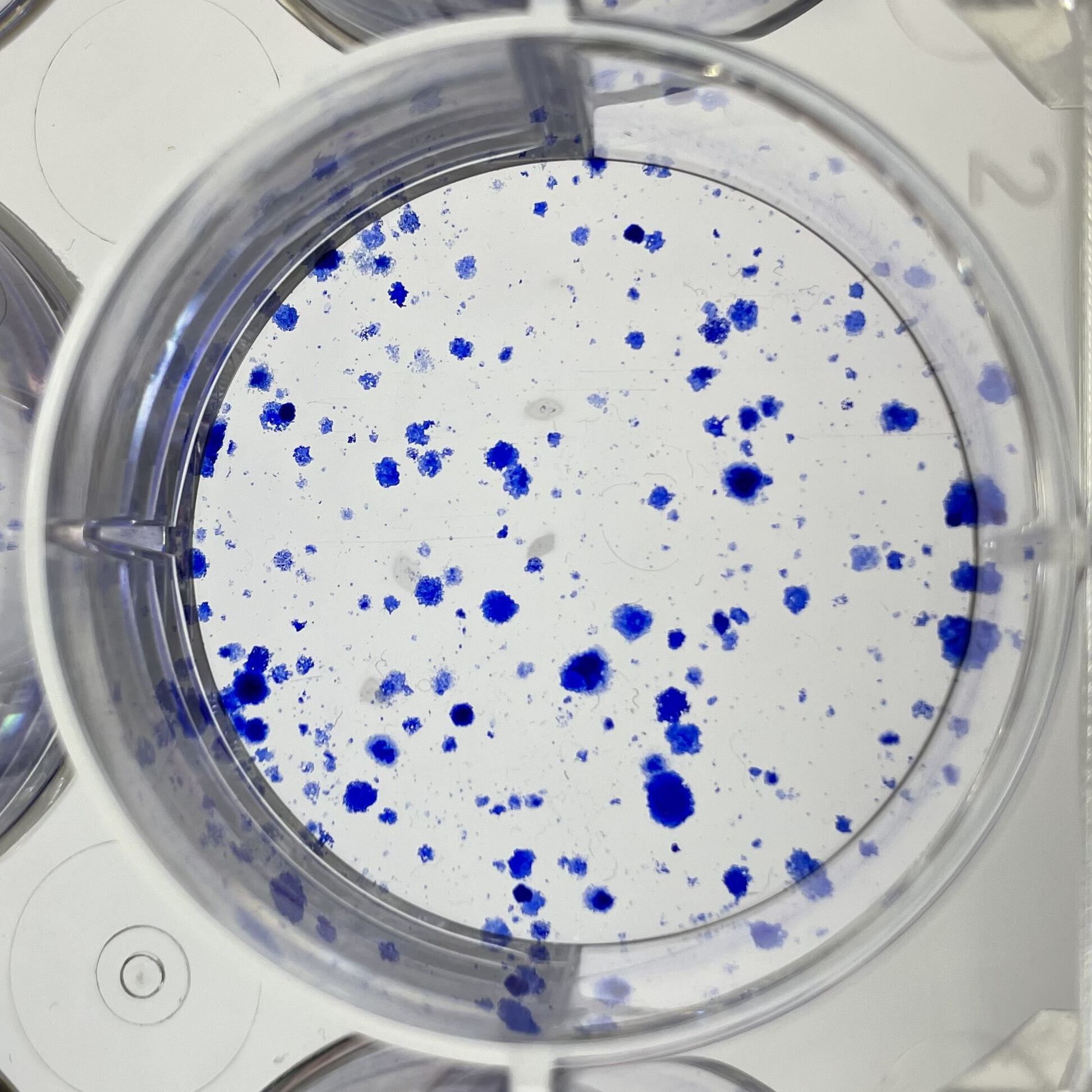

Supplement: Supplementary file 11 — Source Data Fig. 6 [file 44321_2023_3_MOESM11_ESM.zip › Figure 6/Fig 6G-Image data shCtrl-0nM.tif]

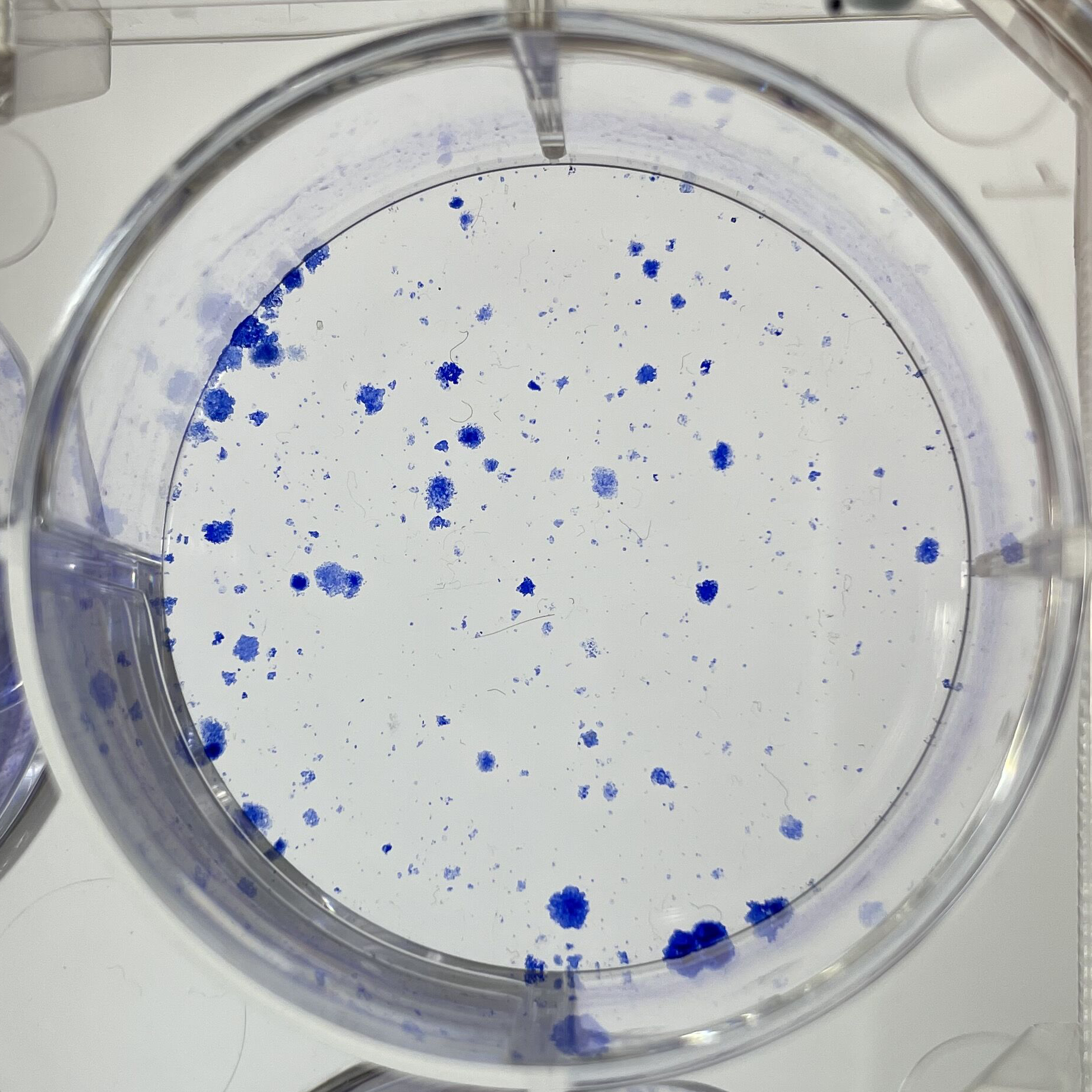

Supplement: Supplementary file 11 — Source Data Fig. 6 [file 44321_2023_3_MOESM11_ESM.zip › Figure 6/Fig 6G-Image data shCtrl-500nM.tif]

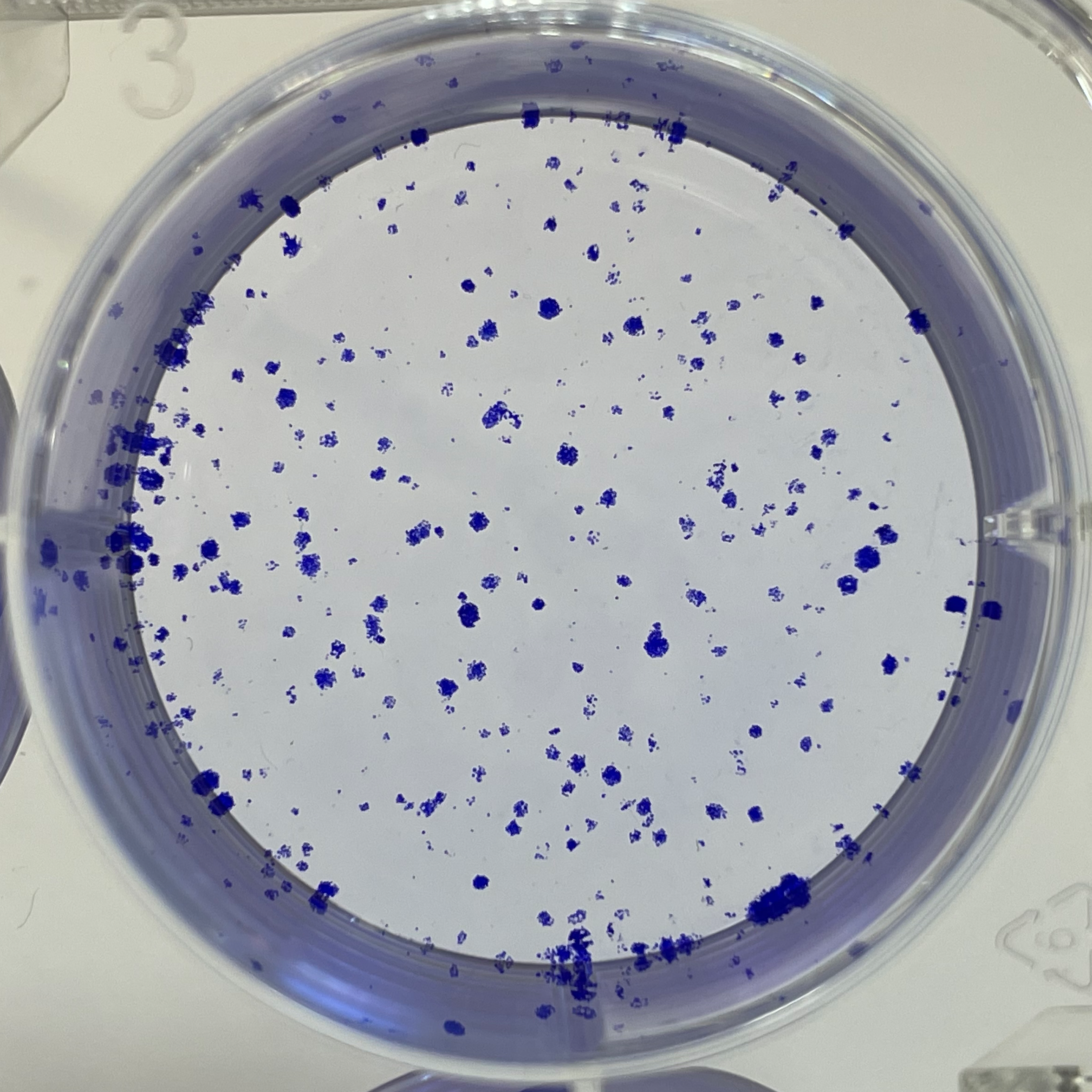

Supplement: Supplementary file 11 — Source Data Fig. 6 [file 44321_2023_3_MOESM11_ESM.zip › Figure 6/Fig 6J-Image data shMYSM1-500nM.tif]

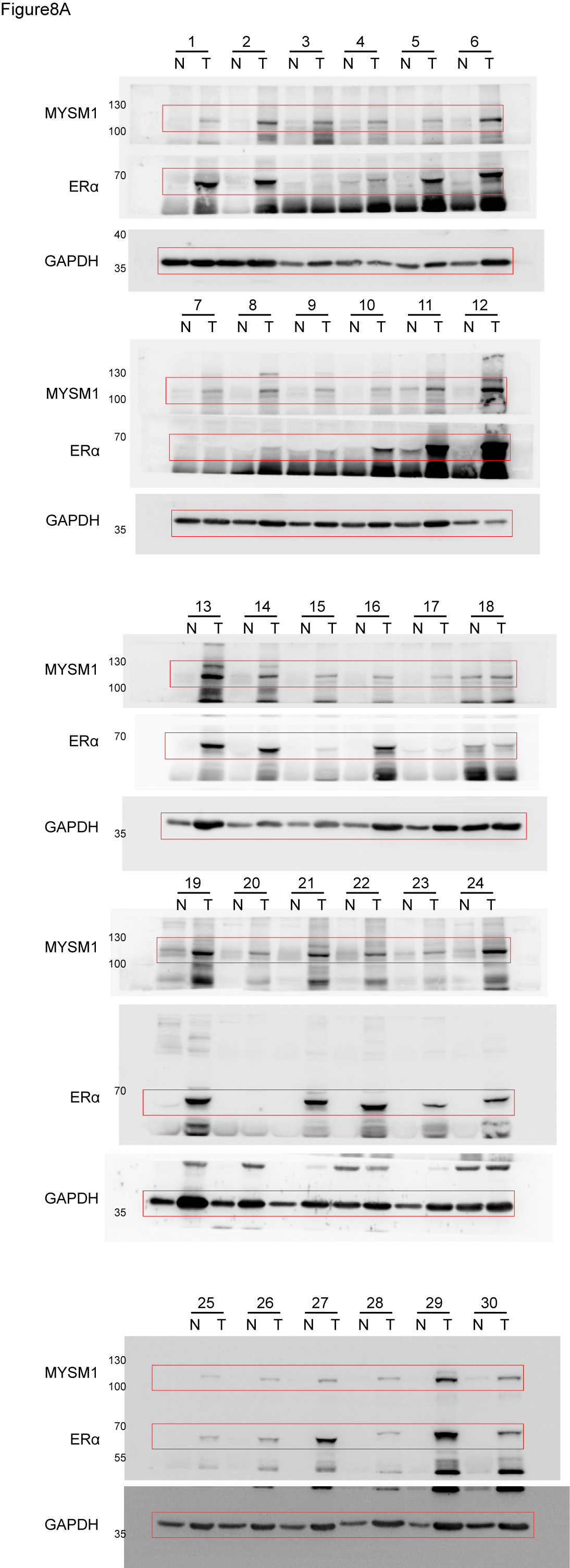

Supplement: Supplementary file 12 — Source Data Fig. 7 [file 44321_2023_3_MOESM12_ESM.zip › Figure 7/Fig 7A-Image data.tif]

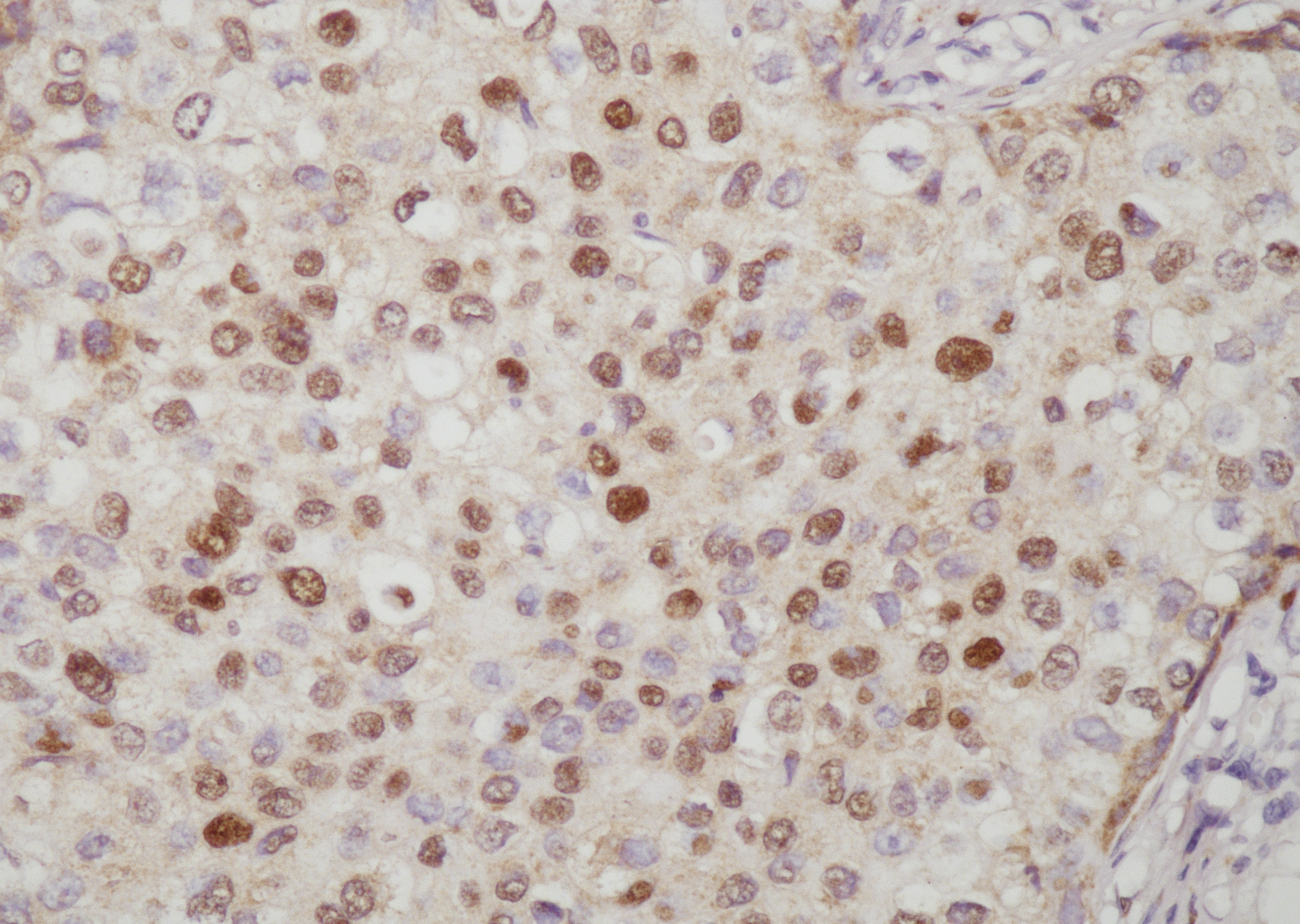

Supplement: Supplementary file 12 — Source Data Fig. 7 [file 44321_2023_3_MOESM12_ESM.zip › Figure 7/Fig 7E-Image data GradeII.tif]

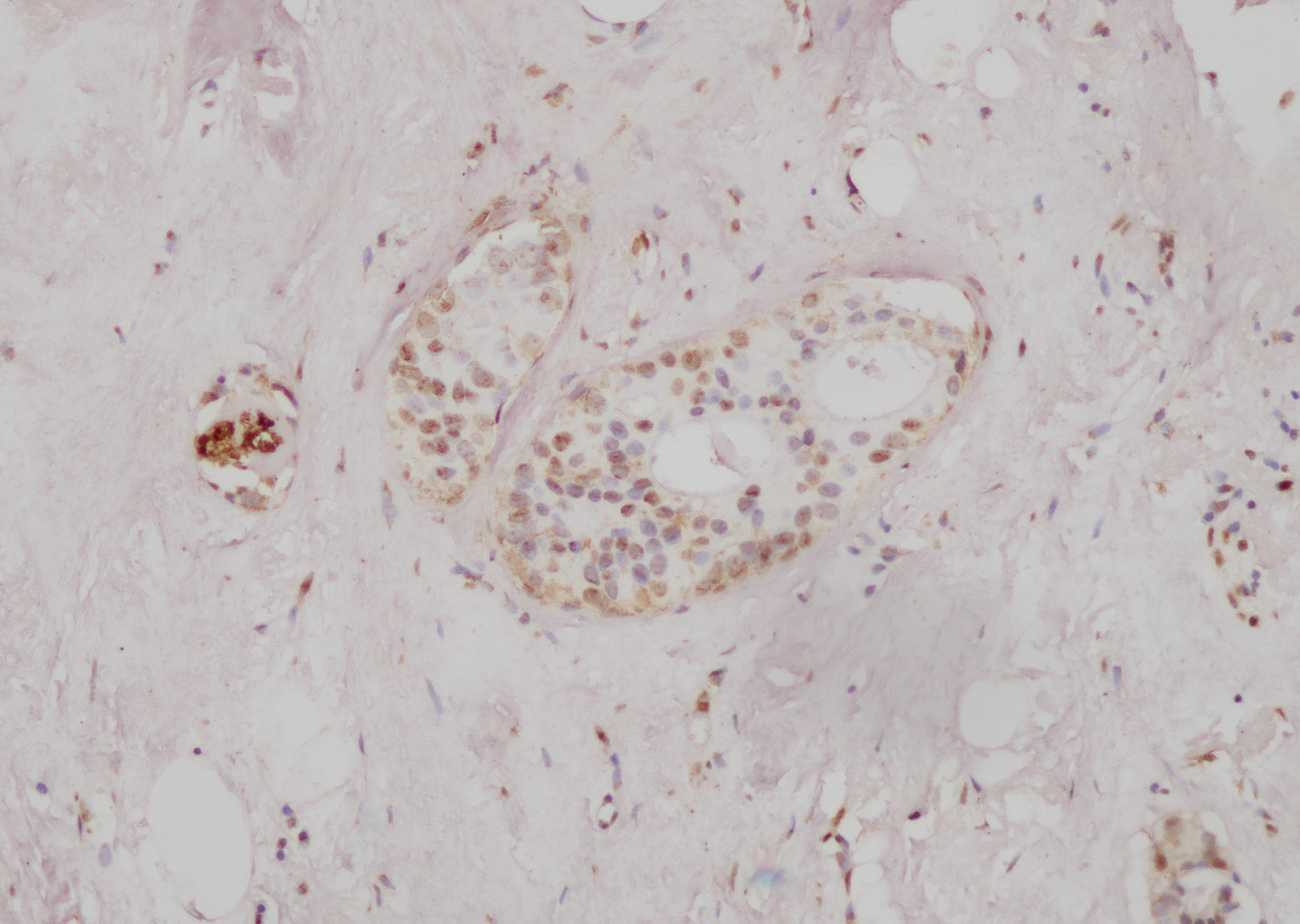

Supplement: Supplementary file 12 — Source Data Fig. 7 [file 44321_2023_3_MOESM12_ESM.zip › Figure 7/Fig 7E-Image data GradeI.tif]

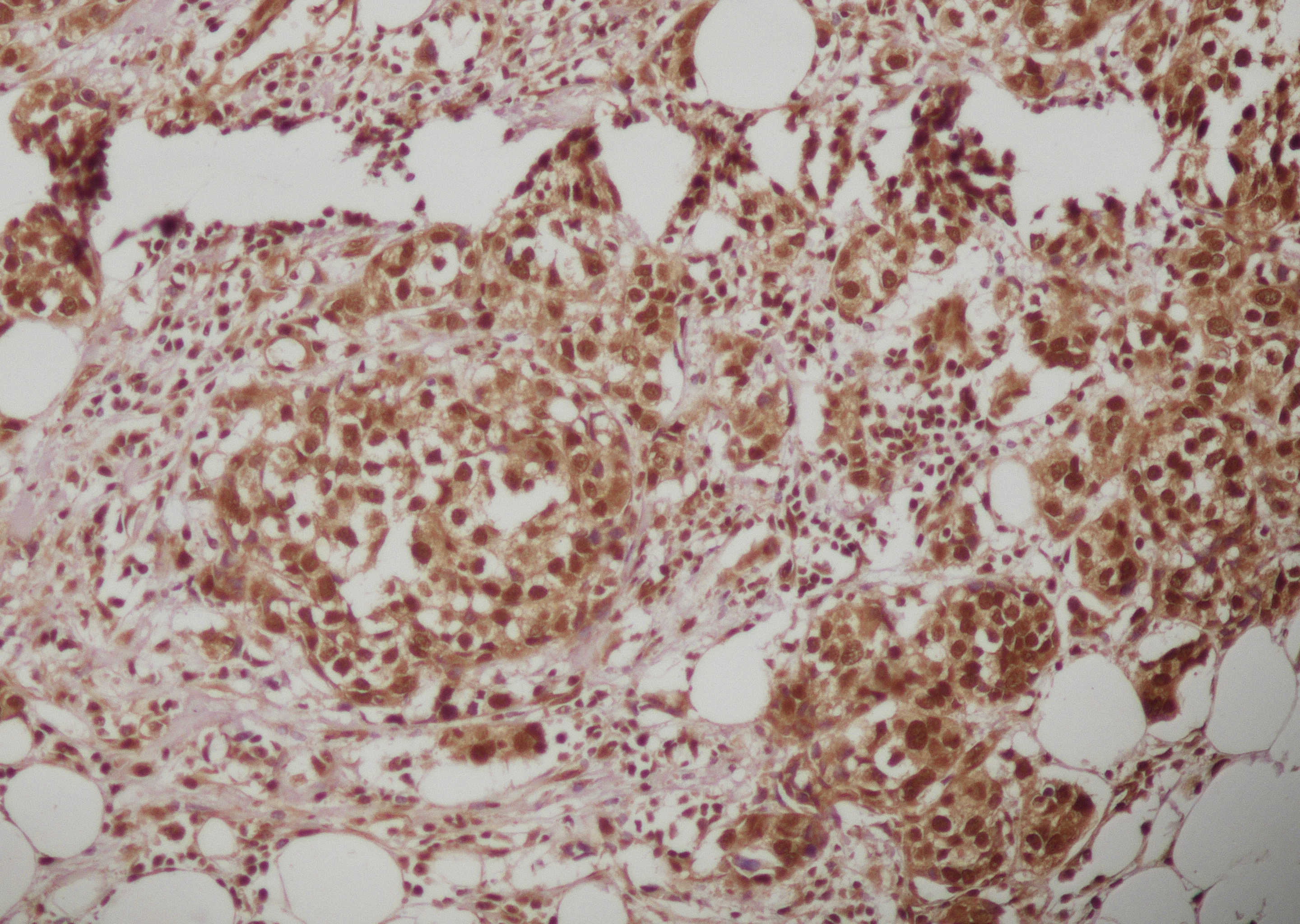

Supplement: Supplementary file 12 — Source Data Fig. 7 [file 44321_2023_3_MOESM12_ESM.zip › Figure 7/Fig 7E-Image data GradeIII.tif]

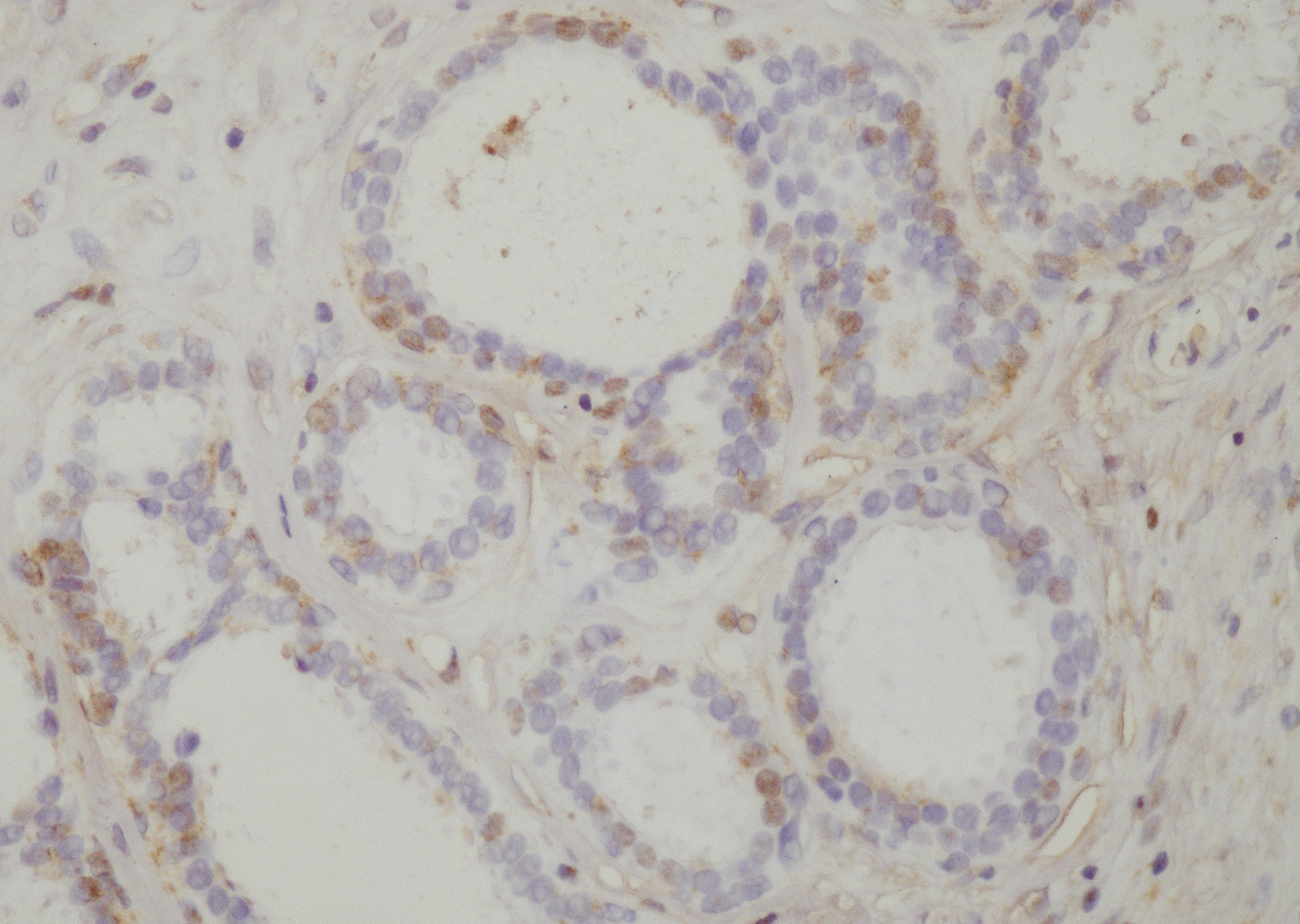

Supplement: Supplementary file 12 — Source Data Fig. 7 [file 44321_2023_3_MOESM12_ESM.zip › Figure 7/Fig 7E-Image data Benign.tif]
