# Supplementary material for: MYSM1 acts as a novel co-activator of ERα to confer antiestrogen resistance in breast cancer
Source: EMBO Mol Med. 2023 Dec 15;16(1):4. doi: 10.1038/s44321-023-00003-z (PMC10883278; doi:10.1038/s44321-023-00003-z)
Supplement: Supplementary file 13 — Expanded View Figures [file 44321_2023_3_MOESM13_ESM.pdf]

# Expanded View Figures

**Figure EV1. MYSM1 interacts with ERα in breast cancer cells.**

(**A**) Evaluation of GFP and ERα level in F1 progeny flies with CG4751 loss of function (lane 3) or MYSM1 gain of function (lane 4) mutants. The lower panels represent merge images. (**B**) The MYSM1-related protein-protein interaction (PPI) networks generated by the STRING online database. (**C**, **D**) Co-immunoprecipitation conducted in T47D cells to detect the association between endogenous MYSM1 and ERα in response to E2 treatment. (**E**) HEK293 cells complemented with ERα or the deletion mutants of MYSM1 were lysed. Complexes precipitated by anti-FLAG were purified and immunoblotted with indicated antibodies. (**F**) Immunofluorescent staining of MYSM1 (anti-FLAG, green) and ERα (anti-ERα, red) in HEK293 cells overexpressing MYSM1 truncated mutants and ERα in the presence of E2. Nuclei were stained with DAPI (blue). Scale bars, 10 μm. Data information: (**C**–**F**): $n = 3$ independent experiments performed in duplicate.

 © The Author(s)

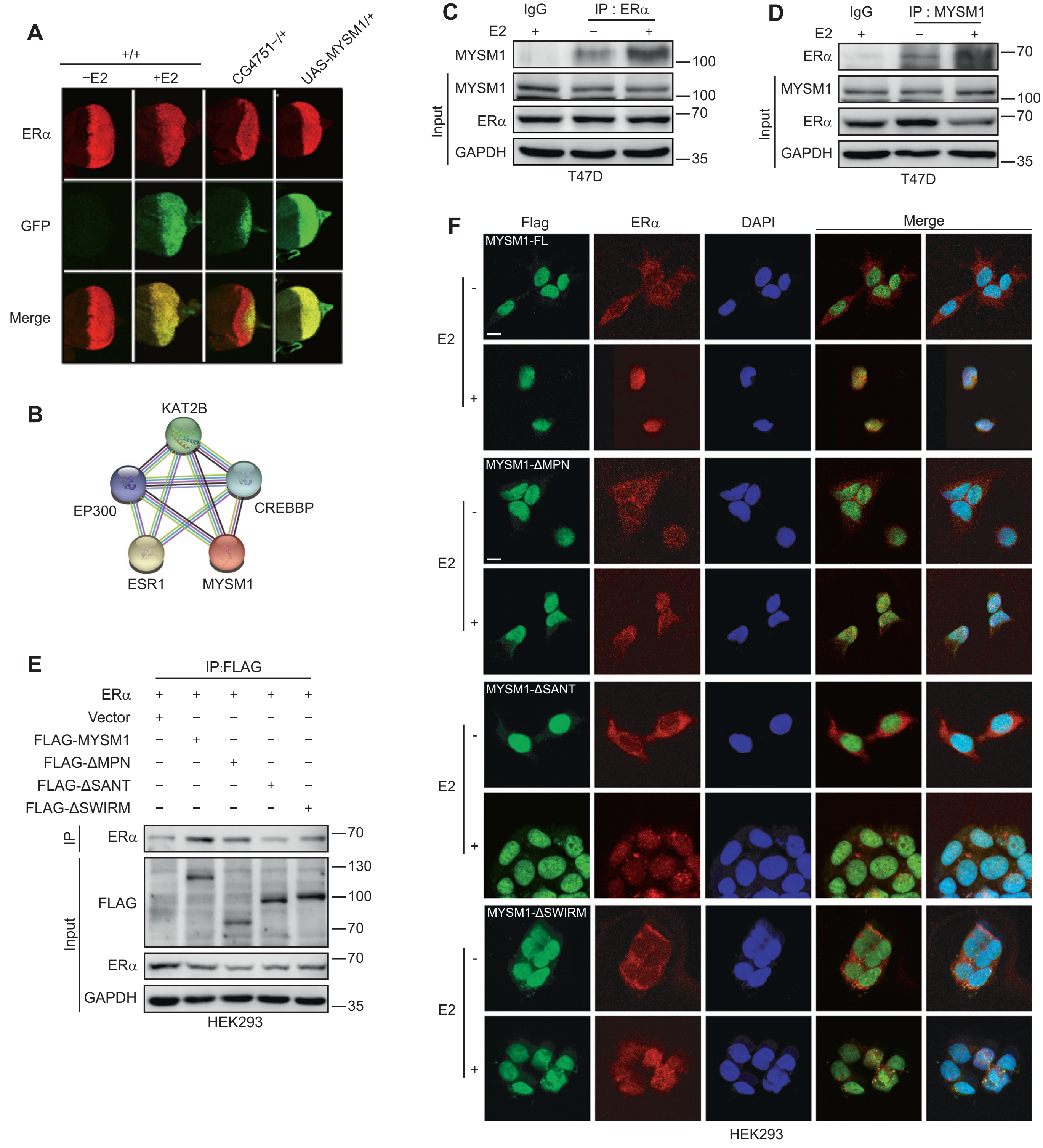
A
+/+
−E2
+E2
CG4751−/+
UAS-MYSM1/+
ERα
GFP
Merge
B
KAT2B
EP300
CREBBP
ESR1
MYSM1
C
IgG
IP : ERα
E2
MYSM1
Input
ERα
GAPDH
100
70
35
T47D
D
IgG
IP : MYSM1
E2
ERα
MYSM1
Input
GAPDH
70
100
35
T47D
E
IP:FLAG
Vector
FLAG-MYSM1
FLAG-ΔMPN
FLAG-ΔSANT
FLAG-ΔSWIRM
IP
FLAG
130
HEK293
F
Flag
ERα
DAPI
Merge
MYSM1-FL
MYSM1-ΔMPN
MYSM1-ΔSANT
MYSM1-ΔSWIRM
E2
HEK293

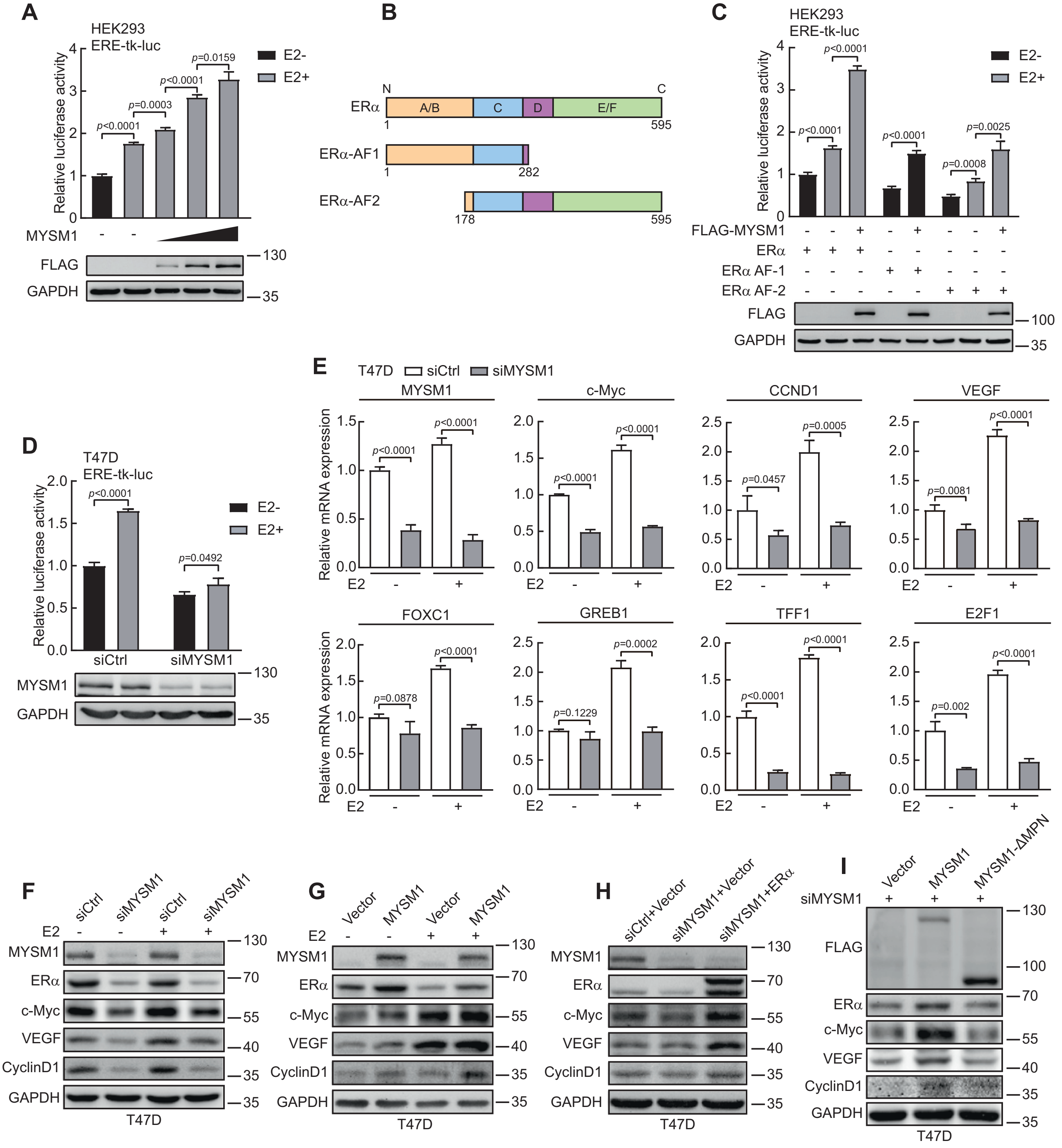
A
HEK293
ERE-tk-luc
Relative luciferase activity
p<0.0001
p=0.0003
p<0.0001
p=0.0159
E2-
E2+
MYSM1
FLAG
GAPDH
130
35
B
ERα
N
C
A/B
C
D
E/F
1
595
ERα-AF1
282
ERα-AF2
178
C
HEK293
ERE-tk-luc
Relative luciferase activity
p<0.0001
p<0.0001
p<0.0001
p=0.0008
p=0.0025
FLAG-MYSM1
ERα
ERα AF-1
ERα AF-2
FLAG
GAPDH
100
35
D
T47D
ERE-tk-luc
Relative luciferase activity
p<0.0001
p=0.0492
siCtrl
siMYSM1
MYSM1
GAPDH
130
35
E
T47D siCtrl siMYSM1
MYSM1
c-Myc
CCND1
VEGF
FOXC1
GREB1
TFF1
E2F1
Relative mRNA expression
E2
p<0.0001
p<0.0001
p<0.0001
p<0.0001
p=0.0457
p=0.0005
p=0.0081
p<0.0001
p=0.0878
p<0.0001
p=0.1229
p=0.0002
p<0.0001
p<0.0001
p=0.002
p<0.0001
F
siCtrl
siMYSM1
siCtrl
siMYSM1
E2
MYSM1
ERα
c-Myc
VEGF
CyclinD1
GAPDH
T47D
G
Vector
MYSM1
Vector
MYSM1
H
siCtrl+Vector
siMYSM1+Vector
siMYSM1+ERα
I
Vector
MYSM1
MYSM1-ΔMPN
siMYSM1
FLAG
130
100
70
55
40
35

◀ **Figure EV2. MYSM1 enhances ERα-mediated gene transcription in mammalian cells.**

(**A**) MYSM1 stimulates ERα-mediated gene transcription in a dose-dependent manner. HEK293 cells were transfected with gradually increased amount of ectopic MYSM1 (0.05 μg, 0.1 μg, or 0.2 μg respectively). MYSM1 expression was examined with anti-FLAG by western blot. (**B**) Schematic representation of ERα, ERα-AF1, and ERα-AF2 plasmids used in luciferase reporter assays. (**C**) Relative luciferase activities in HEK293 cells transfected with ERα full length or truncated mutants harboring ERα AF-1 or ERα AF-2 together with MYSM1 expression plasmid in the presence or absence of E2 (100 nM). The expression of MYSM1 was detected by western blot. (**D**) Effect of MYSM1 knockdown on ERα-induced transactivation. The relative luciferase values in T47D cells were examined after transient transfection of siCtrl or siMYSM1 followed by ERα expression plasmid. (**E**) mRNA levels of several ERα target genes in T47D cells with MYSM1-depleted. (**F**, **G**) Immunoblot of ERα target gene expression using the indicated antibodies in MYSM1-depleted T47D cells (**F**) and MYSM1-overexpressed T47D cells (**G**) with or without E2 (100 nM) treatment for 16-18 h. (**H**) The loss of ERα and its target genes in MYSM1-depleted T47D cells can be rescued by ectopic ERα expression. T47D cells were transfected with siCtrl or siMYSM1 followed by PcDNA3.1/ERα expression plasmid. (**I**) Western blot detecting the protein levels of ERα and its target genes in MYSM1-depleted T47D cells transfected with PcDNA3.1/MYSM1/MYSM1-ΔMPN expression plasmids. Data information: *$P < 0.05$, **$P < 0.01$, ***$P < 0.001$ (mean ± SD; Student's $t$ test; $n = 3$ independent experiments).

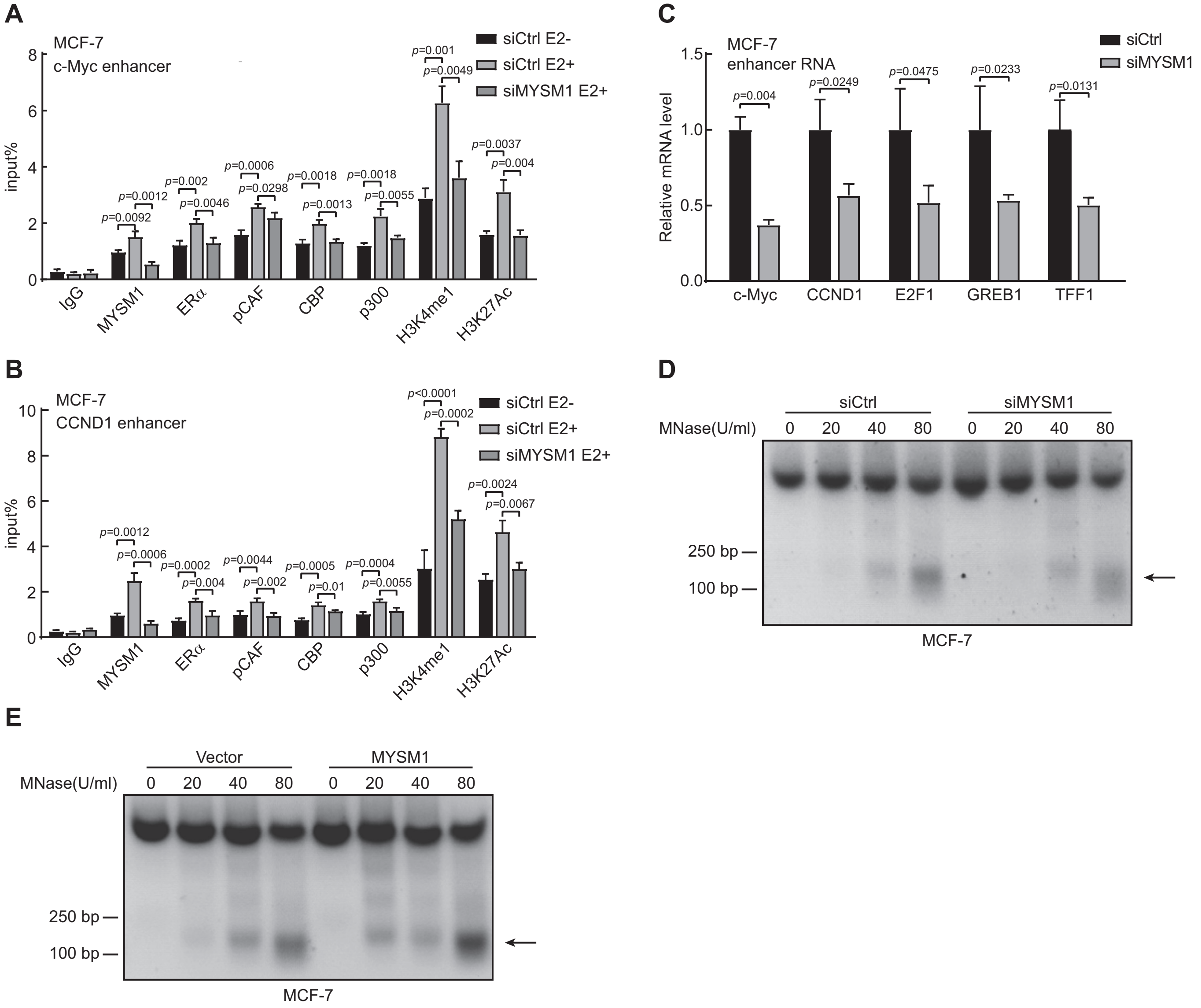


**Figure EV3. MYSM1 enhances the occupation of ERα and HAT complex on E2-regulated enhancers in MCF-7 cells.**

(**A**, **B**) ChIP assays via designated antibodies demonstrating the recruitment of MYSM1, ERα, pCAF, CBP, p300 and the histone modification levels H3K4me1 and H3K27ac at the ERα binding site on *c-Myc* (A) or *CCND1* (B) enhancer in MCF-7 cells upon MYSM1 depletion. (**C**) qPCR analysis demonstrating the eRNA expression of estrogen-induced genes in MCF-7 cells transfected with siCtrl or siMYSM1 in the presence of E2. (**D**, **E**) MNase experiments examining chromatin accessibility after digestion with 0, 20, 40, or 80 U MNase in MYSM1-depletion (**D**) or MYSM1-overexpression (**E**) MCF-7 cells. Data information: (**A**–**C**): $n = 3$ independent experiments. $^{*}P < 0.05$, $^{**}P < 0.01$, $^{***}P < 0.001$, N.S. means no significance (mean ± SD; Student's $t$ test).

 © The Author(s)

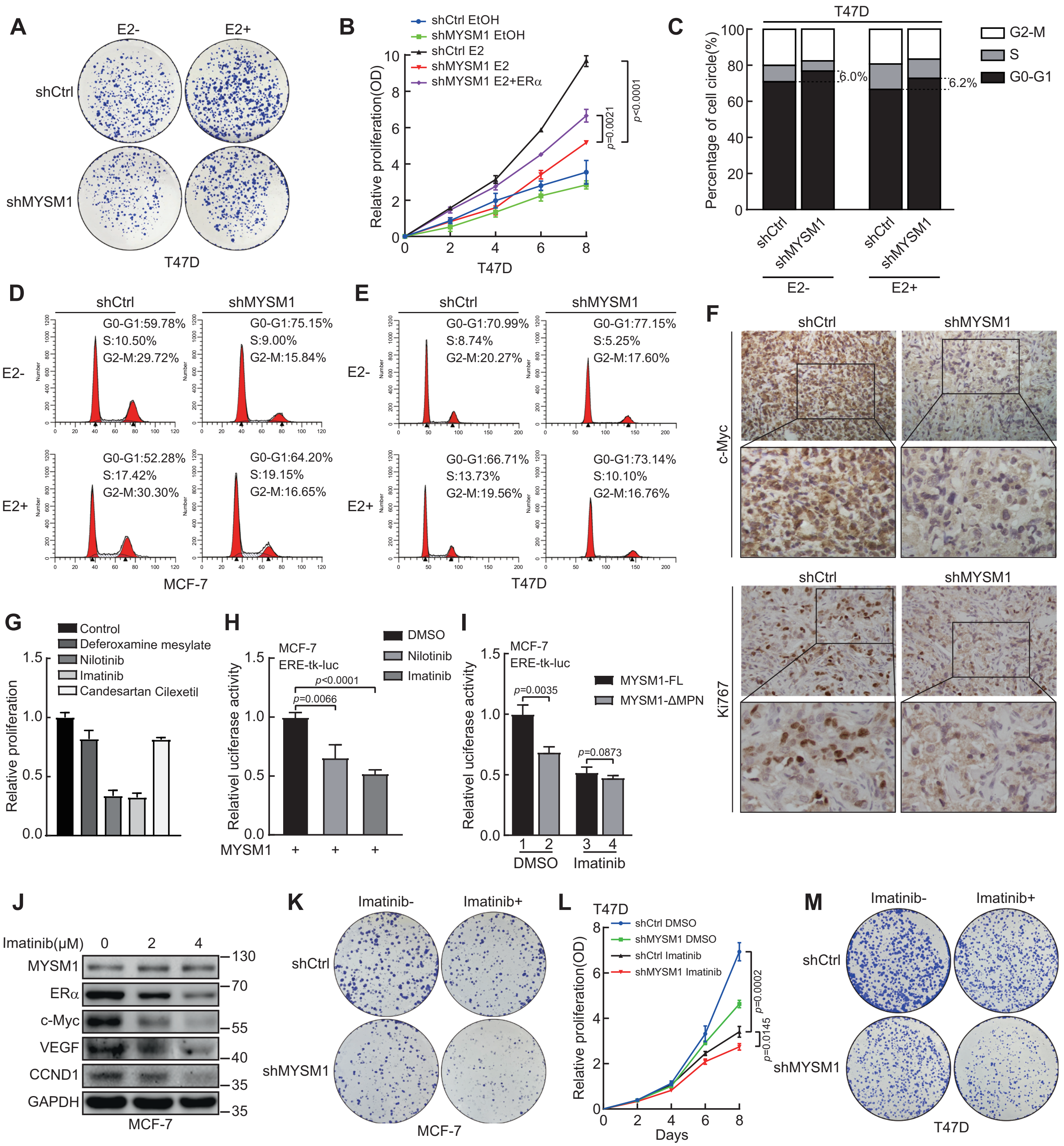
A
E2-
E2+
shCtrl
shMYSM1
T47D
B
shCtrl EtOH
shMYSM1 EtOH
shCtrl E2
shMYSM1 E2
shMYSM1 E2+ERα
Relative proliferation(OD)
T47D
p=0.0021
p<0.0001
C
T47D
Percentage of cell circle(%)
G2-M
S
G0-G1
6.0%
6.2%
shCtrl
shMYSM1
E2-
E2+
D
shCtrl
shMYSM1
E2-
E2+
G0-G1:59.78%
S:10.50%
G2-M:29.72%
G0-G1:75.15%
S:9.00%
G2-M:15.84%
G0-G1:52.28%
S:17.42%
G2-M:30.30%
G0-G1:64.20%
S:19.15%
G2-M:16.65%
MCF-7
E
G0-G1:70.99%
S:8.74%
G2-M:20.27%
G0-G1:77.15%
S:5.25%
G2-M:17.60%
G0-G1:66.71%
S:13.73%
G2-M:19.56%
G0-G1:73.14%
S:10.10%
G2-M:16.76%
T47D
F
c-Myc
Ki767
G
Control
Deferoxamine mesylate
Nilotinib
Imatinib
Candesartan Cilexetil
Relative proliferation
H
MCF-7
ERE-tk-luc
DMSO
Nilotinib
Imatinib
p<0.0001
p=0.0066
Relativel uciferase activity
MYSM1
I
MCF-7
ERE-tk-luc
MYSM1-FL
MYSM1-ΔMPN
p=0.0035
p=0.0873
DMSO
Imatinib
J
Imatinib(μM) 0 2 4
MYSM1
ERα
c-Myc
VEGF
CCND1
GAPDH
130
70
55
40
35
35
MCF-7
K
Imatinib-
Imatinib+
shCtrl
shMYSM1
MCF-7
L
T47D
shCtrl DMSO
shMYSM1 DMSO
shCtrl Imatinib
shMYSM1 Imatinib
p=0.0002
p=0.0145
Relative proliferation(OD)
Days
M
Imatinib-
Imatinib+
shCtrl
shMYSM1
T47D

**Figure EV4. MYSM1 depletion or the small molecule Imatinib docked to MYSM1 suppresses breast cancer cell growth through MYSM1-ERα axis.**

(**A**) Influence of MYSM1 deficiency on T47D cells as illustrated by colony formation. (**B**) Relative proliferation rates of T47D cells carrying shCtrl or shMYSM1 with or without E2 (100 nM) by MTS assay. (**C–E**) Flow cytometric analysis of the cell cycle for MCF-7 (**D**) and T47D (**E**) with MYSM1 depletion. All figures about flow cytometry represented the results of three independent experiments. The proportion of the T47D cell population in different phases are listed in (**C**). (**F**) c-Myc and Ki67 expression by IHC analysis in xenograft tumors derived from MCF-7 (shCtrl) group and MCF-7 (shMYSM1) group. (**G**) Histograms showing the effects of Deferoxamine mesylate (2 μM), Nilotinib (2 μM), Imatinib (2 μM), or Candesartan Cilexetil (2 μM) on MCF-7 cell proliferation. The results are expressed relative to the control (2μM DMSO treated). (**H**) The effect of Nilotinib (2 μM) or Imatinib (2 μM) on luciferase activity in MCF-7 cells transfected with ERα-related dual-luciferase reporter system and MYSM1 expression plasmid. (**I**) The effect of Imatinib (2 μM) on luciferase activity in MCF-7 cells transfected with ERα-related dual-luciferase reporter system, MYSM1-FL or MYSM1-ΔMPN expression plasmid. (**J**) Western blot showing the expression of the indicated proteins in MCF-7 cells treated with different concentrations of Imatinib (0 μM, 2 μM, or 4 μM respectively). (**K**) Colony formation assay showing the effect of Imatinib treatment (1 μM) on shCtrl or shMYSM1 stably expressed MCF-7 cells. (**L**) Growth curve showing the effect of shMYSM1, Imatinib, or their combination (1 μM) on T47D cell proliferation. Total cell viability was assessed every other day by MTS assay. (**M**) Colony formation assay showing the effect of Imatinib treatment (1 μM) on shCtrl or shMYSM1 stably expressed T47D cells. Data information: $^{**}P < 0.01$, $^{***}P < 0.001$ (mean ± SD; Student's $t$ test; $n = 3$ independent experiments).

 © The Author(s)

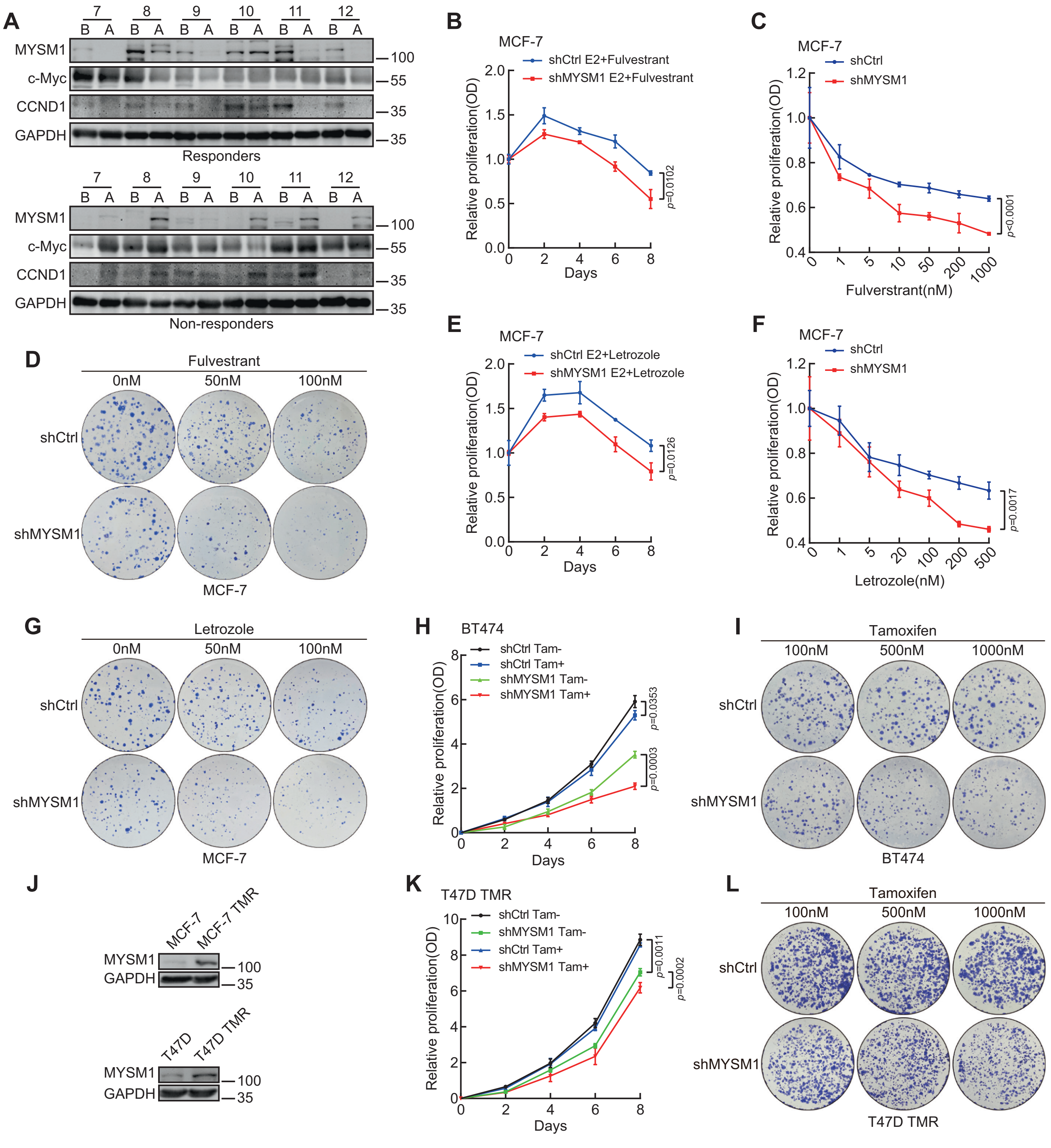
A
7 8 9 10 11 12
B A B A B A B A B A B A
MYSM1
c-Myc
CCND1
GAPDH
100
55
35
35
Responders
Non-responders
B
MCF-7
shCtrl E2+Fulvestrant
shMYSM1 E2+Fulvestrant
Relative proliferation(OD)
Days
p=0.0102
C
MCF-7
shCtrl
shMYSM1
Fulverstrant(nM)
p<0.0001
D
Fulvestrant
0nM
50nM
100nM
shCtrl
shMYSM1
MCF-7
E
MCF-7
shCtrl E2+Letrozole
shMYSM1 E2+Letrozole
p=0.0126
F
MCF-7
Letrozole(nM)
p=0.0017
G
Letrozole
H
BT474
shCtrl Tam-
shCtrl Tam+
shMYSM1 Tam-
shMYSM1 Tam+
p=0.0353
p=0.0003
I
Tamoxifen
100nM
500nM
1000nM
BT474
J
MCF-7
MCF-7 TMR
T47D
T47D TMR
K
T47D TMR
p=0.0011
p=0.0002
L
Tamoxifen
T47D TMR

**Figure EV5. MYSM1 depletion subjects ERα-positive breast cancer cells to endocrine treatment.**

(**A**) MYSM1 and CCND1 protein expression in the “Responders” and “Non-responders” samples before and after AI treatment were examined by western blot. “B” represents cases before AI treatment, “A” represents cases after AI treatment ($n = 6$). (**B**, **E**) Growth curve showing the effect of MYSM1 knockdown on MCF-7 cell proliferation with Fulverstrant (200 nM) (**B**) or Letrozole (100 nM) (**E**) treatment. Total cell viability was assessed every other day by MTS assay. (**C**, **F**) A cellular viability detection in MYSM1-deletion MCF-7 cells that incubated in various concentrations of Fulverstrant (**C**) or Letrozole (**F**) for 7 days. (**D**, **G**) MCF-7 cells with/without MYSM1 knockdown were subjected to colony formation assay under diverse doses of Fulverstrant (**D**) or Letrozole (**G**). Clones were stained with R250 and photographed 2 weeks later. (**H**, **K**) The line chart renders the relative proliferation of the shMYSM1 group compared to the shCtrl group in BT474 (**H**) or T47D TMR (**K**) cells in the stimulation of Tamoxifen (1 μM) or not. (**I**, **L**) The panels show colony-formation assay conducted in BT474 (**I**) or T47D TMR (**L**) cells infected with lentivirus expressing shCtrl/shMYSM1. Cells in each panel were treated with different doses of Tamoxifen for 15 days before fixation and R250 staining. (**J**) Western blot assay detecting MYSM1 protein expression in MCF-7, T47D, and their corresponding Tamoxifen-resistant (TMR) cells. Data information: *$P < 0.05$, **$P < 0.01$, ***$P < 0.001$ (mean ± SD; Student's *t* test; $n = 3$ independent experiments).

 © The Author(s)
